# The energetic and physical concept of gold nanorod-dependent fluorescence in cancer treatment and development of new photonic compounds|review

**DOI:** 10.1039/d3ra05487j

**Published:** 2023-11-02

**Authors:** Dalal Mohamed Alshangiti, Mohamed Mohamady Ghobashy, Haifa A. Alqahtani, Tasneam K. El-damhougy, Mohamed Madani

**Affiliations:** a College of Science and Humanities-Jubail, Imam Abdulrahman Bin Faisal University Jubail Saudi Arabia Madany_2000@yahoo.com; b Radiation Research of Polymer Chemistry Department, National Center for Radiation Research and Technology (NCRRT), Atomic Energy Authority P.O. Box 29, Nasr City Cairo Egypt; c Department of Biology, College of Science, Imam Abdulrahman Bin Faisal University Dammam 31441 Saudi Arabia; d Department of Chemistry, Faculty of Science (Girls), Al-Azhar University P.O. Box 11754, Yousef Abbas Str., Nasr City Cairo Egypt Tasneam92@gmail.com

## Abstract

The optical features of gold nanorods (GNR) may be precisely controlled by manipulating their size, shape, and aspect ratio. This review explores the impact of these parameters on the optical tuning of (GNR). By altering the experimental conditions, like the addition of silver ions during the seed-mediated growth process, the aspect ratio of (GNR) may be regulated. The shape is trans from spherical to rod-like structures resulting in noticeable changes in the nanoparticles surface plasmons resonance (SPR) bands. The longitudinal SPR band, associated with electron oscillations along the long axis, exhibits a pronounced red shift into the (NIR) region as the aspect ratio increases. In contrast, the transverse SPR band remains relate unchanged. Using computational methods like the discrete dipole approximation (DDA) allows for analyzing absorption, scattering, and total extinction features of gold (G) nanoparticles. Studies have shown that increasing the aspect ratio enhances the scattering efficiency, indicating a higher scattering quantum yield (QY). These findings highlight the importance of size, shape, and aspect ratio in controlling the optical features of (GNR) providing valuable insights for various uses in nanophotonics and plasmonic-dependent fluorescence in cancer treatment and developing new photonic compound NRs.

## Abstract 2

The optical features of gold nanorods (GNR) may be precisely controlled by manipulating their size, shape, and aspect ratio.

## Graphical abstract

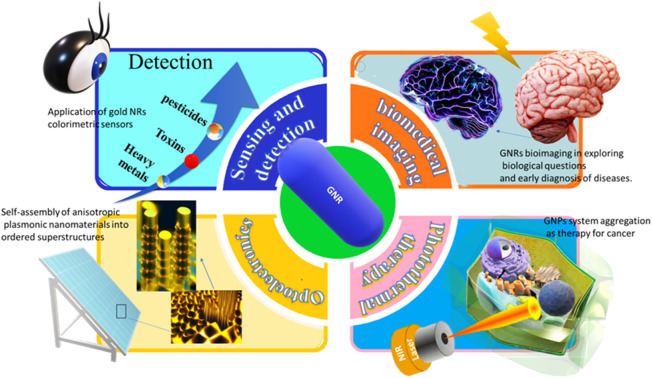

## 1 Introduction

In the realm of nanomaterials, few entities shine as brilliantly as gold nanorods (GNRs). These remarkable structures, with dimensions on the nanometer scale, have garnered immense attention in recent years for their exceptional optical properties and versatile applications. As we delve into the intricate world of nanophotonics and beyond, GNRs emerge as dazzling stars guiding our path towards novel technological frontiers. Gold nanorods are a testament to the astonishing transformations that materials can undergo at the nanoscale. Unlike their bulk counterparts, GNRs exhibit tunable plasmon resonances—a phenomenon where free electrons oscillate collectively in response to incident light. This unique property endows them with the power to interact with and manipulate light in ways previously unattainable, making them a subject of intense scientific exploration and innovation. Nanotechnology technique is a helpful method for different applications^1,2^ it can be prepared from template methods,^3^ as semi-permeable membrane,^4^ blend polymer^5^ nanoparticles aims to use as green renewable resources to protect the environment from harmful effects.^6–8^

### 1.1 Pioneering the gold nanotechnological frontier in photothermal therapy

The study of nanomaterials continues to push the boundaries of technology, offering solutions to overcome constraints in various fields. As researchers delve deeper into the features and uses of nanomaterials, we can expect further breakthroughs that revolutionize industries and improve the quality of life. Moreover, (G)nanomaterials have been utilized in photothermal therapy (PTT), a promising cancer treatment modality. When exposed to (NIR) light, (GNP) can efficiently convert light into heat, destroying cancer cells through localized hyperthermia while minimizing damage to healthy tissue. (G)nanometric devices offer tremendous potential in targeted drug administration and other biological uses. Their unique features and ability to functionalize and engineer them for related purposes make them a valuable tool in advancing precision medicine and improving patient outcomes.

### 1.2 Unique properties of gold nanoparticles with multimodal approaches and synergistic effects

Combining biosensing and bioimaging capabilities, (G)nanometric devices offer versatile platforms for molecular diagnostics, real-time monitoring of biological processes, and personalized medicine. Their unique feature drives innovation and contributes to developing advanced biomedical technologies. Combining (G) particle nanostructures with other therapeutic modalities, like chemotherapy or radiation therapy, can lead to synergistic impacts. For instance, (GNP) may be loaded with chemotherapy drugs and guided to the tumor site. Upon reaching the tumor, the (GNP) can release the drug payload and be subsequently activated by light to induce localized hyperthermia. This multimodal approach enhances treatment efficacy by combining the advantages of various therapeutic modalities. It is well-known for its high electrical conductivity, making it a valuable material in various uses that require excellent electrical conductivity; (G) is generally considered non-magnetic, meaning it does not exhibit strong magnetic properties. In its pure form, (G) is classified as a diamagnetic material, which implies that it weakly repels magnetic fields. In certain instances, (GNP) may be engineered to possess magnetic properties. For example, by coating (GNP) with magnetic materials like iron oxide, the resulting composite NPs can exhibit magnetism because of the magnetic feature of the iron oxide component. These magnetic (GNP) find uses in areas like magnetic resonance imaging (MRI), drug delivery, and magnetic separation techniques. (G)exhibits unique contrast features, making it valuable in various imaging techniques and contrast-enhanced uses. In imaging modalities like X-ray computed tomography (CT) and electron microscopy, (G) is commonly used as a contrast agent because of its high atomic number. The high atomic number of (G) results in strong X-ray absorption and scattering, resulting in excellent contrast in CT scans and enhanced visibility of (G) labeled structures or particles in electron microscopy. When (G) nanometric materials are coupled with SPIONs, they create a multifunctional platform with combined properties. The (G) component provides unique optical properties, like plasmonic resonance, which may be used for imaging and sensing. (GNP) can also serve as carriers for therapeutic agents or vehicles for drug delivery because of their biocompatibility and versatile surface chemistry.^9–12^ The resulting graphene oxide (GO-GNR) hybrid system may be utilized for selective photothermal therapy, where laser irradiation of the hybrid material generates localized heat, leading to targeted cell destruction in cancer treatment,^13–16^ silica NPs for example, in imaging uses, the combination of silica NPs and (GNR) can lead to improved contrast and sensitivity. The (GNRs) contribute to enhanced optical imaging because of their strong absorption and scattering properties, while the silica shell can provide stability and biocompatibility for targeted imaging approaches.^13,17–19^ In summary, combining (GNR) and QDs offers a versatile hybrid system with enhanced optical features and potential imaging, sensing, and theragnostic uses. The integration of the unique plasmonic feature of (GNR) with the tunable emission feature of QDs provides opportunities for advanced imaging, sensitive detection, and multifunctional platforms for biomedical uses^17,20,21^ and colloidal (G) nanoparticles, including (GNR), have found diverse uses in various fields. Their distinct optical feature makes them valuable in biomedical imaging, where they can play as contrast agents for techniques like dark-field microscopy, electron microscopy, or surface-enhanced Raman spectroscopy (SERS). Additionally, the colloidal (GNP) surface may be functionalized with biomolecules, like antibodies or aptamers, allowing for related targeting and recognition in biosensing uses.^20,22–25^ The adaptability and versatility of GNP in cancer treatment have led to advancements in targeted therapies, imaging techniques, and multimodal approaches. Ongoing research continues to explore the potential of GNP in improving cancer treatment outcomes and developing novel therapeutic strategies.^26–28^ The synthesis of (GNP) offers a high degree of control over their sizes, shapes, and physicochemical properties. By adjusting the synthesis processes and parameters, researchers can tailor the characteristics of GNP to suit related uses. Some key factors may be modified during the synthesis to achieve the desired GNP feature.^29–31^ GNP exhibits a unique optical phenomenon called (LSPR), which arises from the collective oscillation of free electrons on the surface of the nanoparticles. This property gives rise to the distinct optical feature of (G), including its color and strong light-matter interactions.^32–34^

### 1.3 Localized surface plasmon resonance in gold NPs for biomedical applications

While (LSPR) in (GNP) contributes to their distinct optical properties, it is paramount to note that LSPR primarily affects the absorption and scattering of light rather than the interaction with diffuse electromagnetic waves used in imaging methods like computed tomography (CT) or confocal microscopy.^35,36^ Additionally, the absorbed electromagnetic radiation may transform energy from light into heat; for this reason, (GNP) impacts (PTT) of cancer treatment.^37–39^ The LSPR band of GNP is located in the ultraviolet-visible-near Infrared UV-vis-NIR spectrum, albeit their precise location and number depend on the size, aspect ratio, shape, and aggregation state of the NPs.^37,38,40,41^ One of the notable advantages of (GNR) is the positioning of their (LSPR) in the (NIR) region, typically ranging from 650 nm to 900 nm. This related spectral range is noticeable because it corresponds NRs to the “biological window,” where the absorption of water, hemoglobin, and other biomolecules is related low. This minimized absorption by biological tissues allows NIR light to penetrate deeper into the tissue, enabling enhanced imaging and therapeutic uses.^42–46^ Although the bio-inertia of colloidal (G) makes it unique, capping agents are essential for biological uses.^47,48^ A well-designed coating can improve the stability and longevity of (GNR) in biological environments. It can protect the (GNR) from degradation, enzymatic reactions, or clearance by the immune system, thereby prolonging their existence and impact in the targeted area.^49–51^ Additionally, selecting suitable coating agents enables the loading of therapeutic compound NRs and active targeting functions, increasing cytocompatibility and achieving targeted drug delivery.^52,53^ Small molecule lipoic acid (LA), present in the human body naturally, has been shown to own both ROS scavenging and chelating metal features.^52,54,55^ From a structural standpoint, lipoic acid is distinguished by an intracycle disulfide group that can interact with the (G) surface through the (G)-sulfur chemistry and terminal ionizable carboxylic groups that confer good overall hydrophilicity.^56^ Small molecules may impact stabilize (GNR)-based drug delivery systems. However, a biocompatible polymeric covering is typically used to improve cytocompatibility and drug loading capacity.^56^ As GNP capping agents, polymeric hydrophilic or amphiphilic materials have received much attention. They include natural and manufactured polymers, like proteins and polysaccharides, and synthetic polymers, like polyethylene glycol PEG-SH and polyamine acidic structures. The linear polysaccharide gellan gum (GG) has a negative net charge. It is composed of tetrameric repeating saccharide molecules like ((d-glucose)–(d-glucuronic acid)–(d-glucose)–(l-rhamnose)).^57^ Sphingomonas elodea produces it, and it is H_2_O soluble and biodegradable *in vivo*.^58^ (GG) is a polysaccharide compound approved by the Food and Drug Administration (FDA) as a food additive.^59,60^ It is distinguished as a polysaccharide by carbohydrate components abundant in hydroxyl groups capable of forming hydrogen bonds between NRs with various classes of molecules.^61,62^ Because the greatest absorption band alters with the refractive index (RI) of the local material, (GNRs) are thought to be excellent candidates for sensing biological uses.^63,64^ This allows for incredibly accurate sensing. Additionally, a laser pulse at the absorbance band wavelength can stimulate NRs with near-IR absorption peaks to produce heat, thus enabling the selective thermal death of malignant tissues.^65–68^ The Murphy's (GNR) synthesis technique is mentioned in Table 1.^69,70^

**Table 1:** Preparation of (G) seed

Reagents	Chemical structure	Quantity (mL)
0.01 M HAuCl_4_·3H_2_O	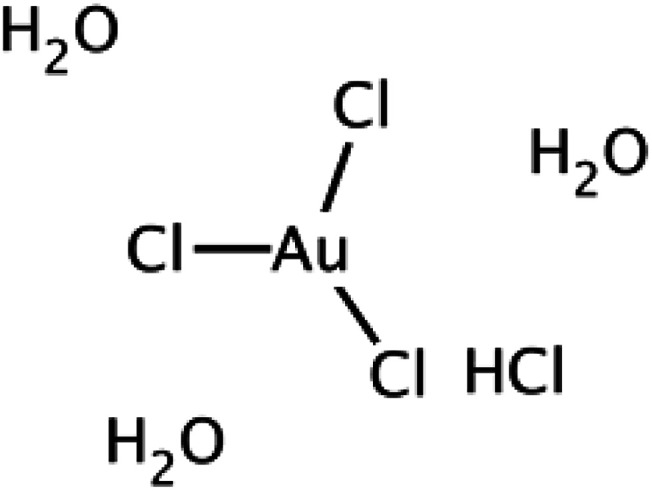	0.250
0.1 M CTAB (cetyltrimethylammonium bromide)	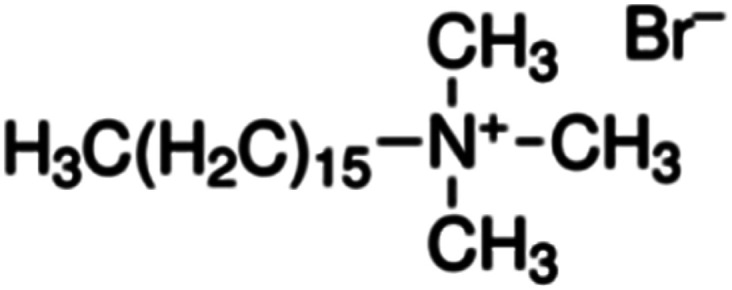	7.5
0.01 M NaBH_4_	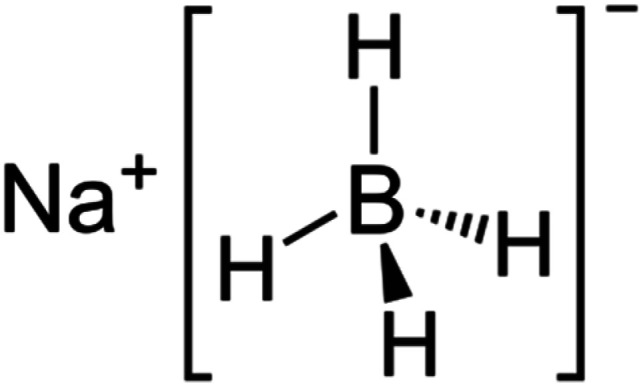	0.6

### 1.4 Synthesis of gold nanorods

**Fig. 1:**
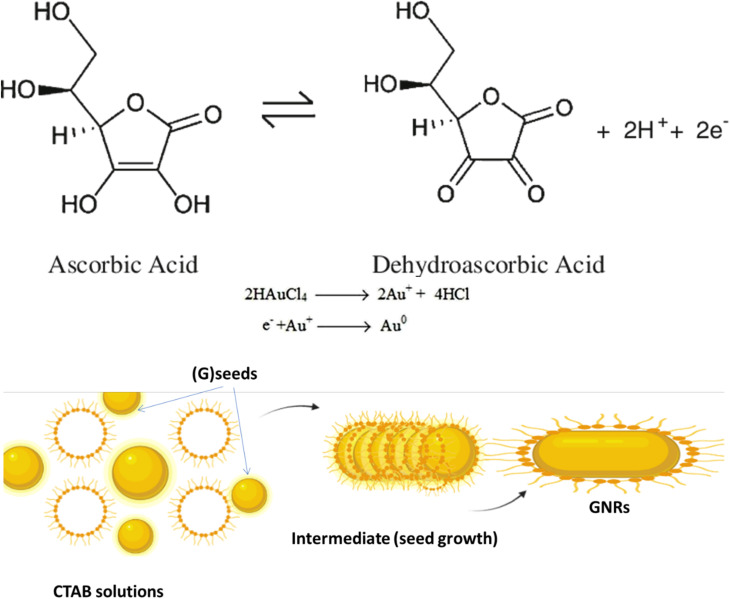
Illustrates the reduction mechanism of (G) ions (Au^3+^) to elemental (G) (Au^0^) by (AA) and the role of CTAB in controlling the anisotropic (GNR) growth. CTAB molecules on the seed surface control the deposition and arrangement of (G) atoms during the reduction process, producing rod-shaped nanoparticles. By regulating the concentration and interaction of CTAB and the reaction conditions, it becomes possible to control the size and aspect ratio of the (GNR) synthesized using Murphy's (GNR) synthesis technique. The interplay among (AA), CTAB, and (G) ions enables the controlled growth of anisotropic (GNPs).

The synthesis of (GNR) typically involves the reduction of (G) salts using a reducing agent like sodium borohydride (NaBH_4_). During the synthesis, the surfactant (CTAB) stabilizes the seed particles, preventing aggregation. Adding related ingredients, including a trace amount of silver, is necessary to regulate the shape and size of the (GNR). Without CTAB, the NPs that form would typically be spherical. CTAB acts as a template for the production of rod-shaped particles by directing the growth and alignment of (G) particles during synthesis. This synthesis method uses (AA) as a reducing agent in addition to CTAB. Although it is a weaker reducing agent than NaBH_4_, (AA) can still impact reduce (G) ions at the seed particles surface in the existence of the CTAB template. This combination allows for the controlled (GNR) growth. Fig. 1 likely illustrates the experimental setup or results of the synthesis process, showing the production of (GNR) using the CTAB template and (AA) as the reducing agent. Finally, the combination of NaBH_4_, CTAB, and (AA) in the synthesis process enables the production of GNR with controlled size and shape. CTAB acts as a stabilizer and template, while (AA) reduces (G) ions on the seed surface, resulting in the desired rod-shaped nanoparticles.

Table 1 introduces preparing the seed solution for synthesizing (GNPs) using Murphy's (GNR) synthesis technique. The table lists the reagents, their chemical structures, and the quantities used in milliliters (mL).

The reagents and their quantities mentioned in Table 1 are as follows:

(1) 0.01 M HAuCl_4_·3H_2_O refers to a solution of (HAuCl_4_) with a concentration of 0.01 mol L^−1^. The corresponding amount used is 0.250 mL.

(2) 0.1 M CTAB (cetyltrimethylammonium bromide): CTAB is a surfactant that significantly stabilizes the seed particles and directs their growth. It is used at a concentration of 0.1 mol L^−1^, with a quantity of 7.5 mL.

(3) 0.01 M NaBH_4_: sodium borohydride (NaBH_4_) is a reducing agent that aids in the reduction of (G) ions to form (GNPs). It is used at a concentration of 0.01 mol L^−1^, with a quantity of 0.6 mL.

The reduction mechanism of (Au^0^) by (AA) (AA) in the existence of (CTAB) may be described as follows:

*Seed formation*: CTAB acts as a stabilizing agent for the (G) seed particles. CTAB molecules adsorb onto the (G) seeds surface, forming a protective layer that prevents their aggregation.

*Reduction of (G) ions*: In the existence of CTAB, (AA) acts as a reducing agent for (G)ions (Au^3+^). The reduction occurs at the (G) seeds surface, where (AA) transfers electrons to (Au^3+^), forming (Au^2+^), then (AA) transfers electrons to (Au^2+^), forming (Au^+^). (AA) transfers electrons to (Au^+^), forming (Au^0^) indicates that the growth and nucleation of G by AA is slow. The reduction process may be related to slow AA compared to other reducing agents. This slow kinetics may be attributed to the nature of AA as a weaker reducing agent compared to other reagents commonly used in (G) nanoparticle synthesis.^71^ However, the use of UV irradiation can assist in accelerating the reduction process and promoting the growth and nucleation of (GNPs).^72^

*Anisotropic growth*: CTAB plays a considerable role in controlling anisotropic (GNR) growth. CTAB molecules are structurally amphiphilic, having both hydrophilic and hydrophobic regions. The CTAB molecules adsorbed on the (G)seed surface form a template that directs the (G) atoms growth in a related direction.

The +Ve charged hydrophilic part of CTAB interacts with the negatively charged (G) atoms, while the hydrophobic part exits NRs outward, providing a repulsive barrier among the growing (GNR). This spatial arrangement guides the preferential (G) growth along the longitudinal axis, producing NR with an elongated shape. The current review aims to provide an overview of the unique optical properties of gold nanoparticles, with a specific focus on gold nanorods. The key objectives are:

(1) Explain how gold nanorods exhibit distinctive optical characteristics attributed to localized surface plasmon resonance, highlighting their potential applications in various fields such as sensing, imaging, and photothermal therapy.

(2) Emphasize the importance of controlling the aspect ratio and shape of gold nanorods to precisely tailor their optical features, particularly the longitudinal surface plasmon resonance band, which can be redshifted into the near-infrared region for improved tissue penetration.

(3) Introduce different synthesis methods, including seed-mediated growth, template methods, and photochemical methods, along with the use of silver ions to control the nanorods' shape and aspect ratio.

(4) Underscore the influence of size, shape, and aspect ratio on the optical properties of gold nanorods and how computational methods can model these properties to optimize their suitability for specific applications.

(5) Highlight the promising applications of gold nanorods in areas such as photothermal therapy, drug delivery, and biosensing while acknowledging the need for further research to enhance biocompatibility and targeted delivery.

(6) Explore the potential of using gold nanorods as a versatile delivery platform for combining multiple therapeutic modalities, such as chemotherapy and photothermal therapy, to achieve synergistic effects in addressing challenges like drug resistance and improving treatment outcomes.

## 2 Exploring the characterization and diverse preparation methods of (GNR)

**Fig. 2:**
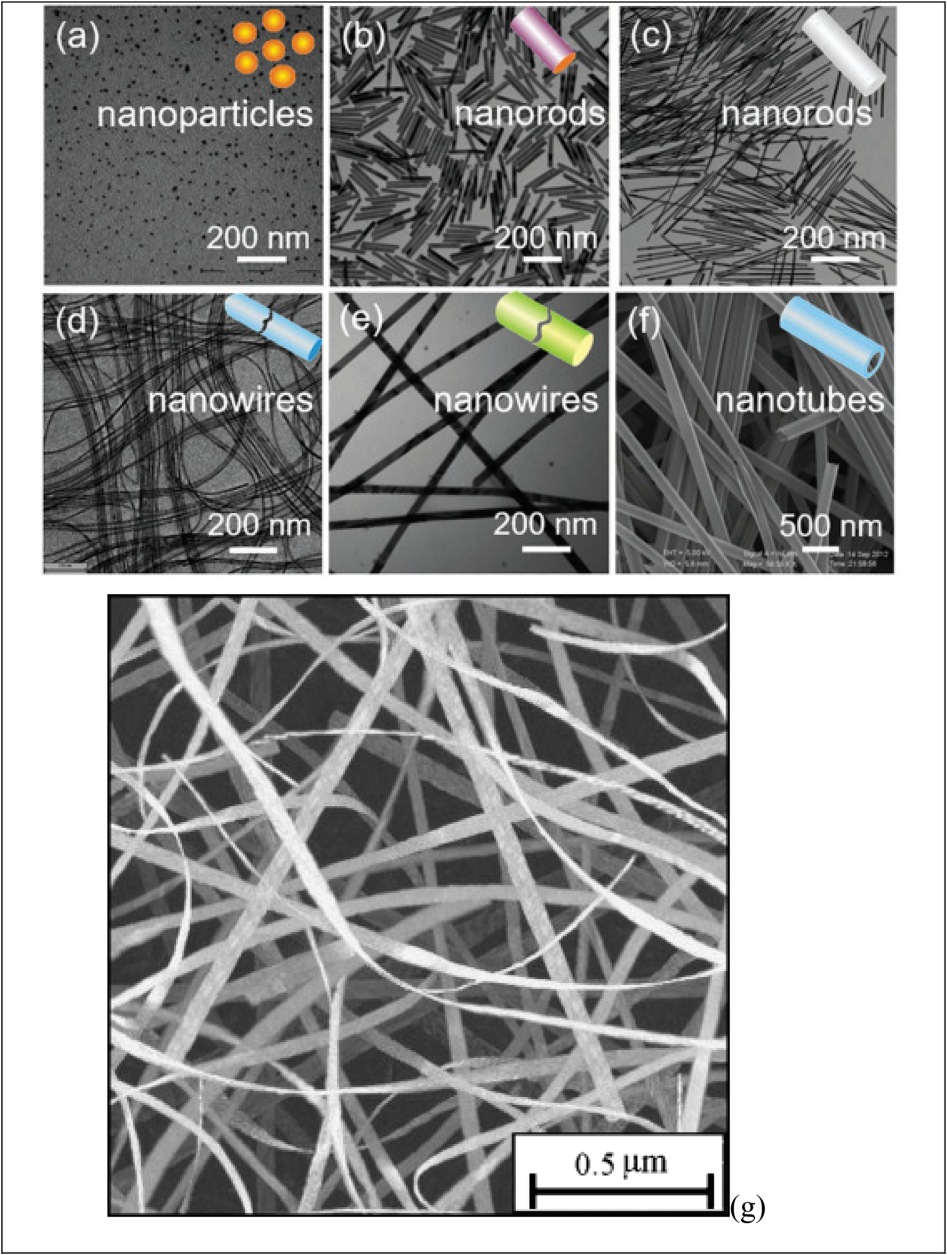
Display various shape of obtained one-dimensional (1D) nanomaterials: (a) NPs, (b, c) NRs, (d–f) nanowires and (g) nanobelt. Ref. 73 Copyright 2021 Elsevier.

Fig. 2 shows diverse examples of one-dimensional nanomaterials. Each shape offers distinct features and uses, making them valuable in various fields, including electronics, energy, medicine, and materials science. (a) NPs are spherical or near-spherical structures with a uniform size distribution. They have a high surface area-to-volume ratio and are typically a few nanometers to a few hundred nanometers in diameter. Examples of NPs include (G) nanoparticles, Ag nanoparticles, and quantum dots. (b) NRs are elongated structures with a cylindrical shape. They own a relatively high aspect ratio, meaning their length is significantly greater than their diameter. NRs may be synthesized with various materials. (c) Nanowires are one-dimensional structures with long, thin, and wire-like shapes. They have a uniform diameter, typically in the nanometer range, while their length can vary from a few micrometers to several centimeters. Nanowires may be made from various materials, including semiconductors like silicon, metal oxides, or carbon nanotubes. (d) Nanotubes are hollow cylindrical structures composed of rolled-up sheets of materials, like carbon or metal oxides. They own a tubular shape and can hold single-walled or multi-walled configurations. Nanotubes exhibit unique electrical, mechanical, and thermal properties, making them valuable for various uses. (e) Nanofibers are long, thin fibers with diameters in the nanometer range. They may be produced from multiple materials, including polymers, metals, and ceramics. Nanofibers own high aspect ratios and a large surface area, making them useful in filtration, tissue engineering, and energy storage. (f) Nanobelts are flat, elongated nanostructures with rectangular or ribbon-like shapes. They own a width in the nanometer range and a larger length-to-width aspect ratio. Nanobelts are typically made of semiconductor materials and can exhibit unique electrical and optical properties.

**Fig. 3:**
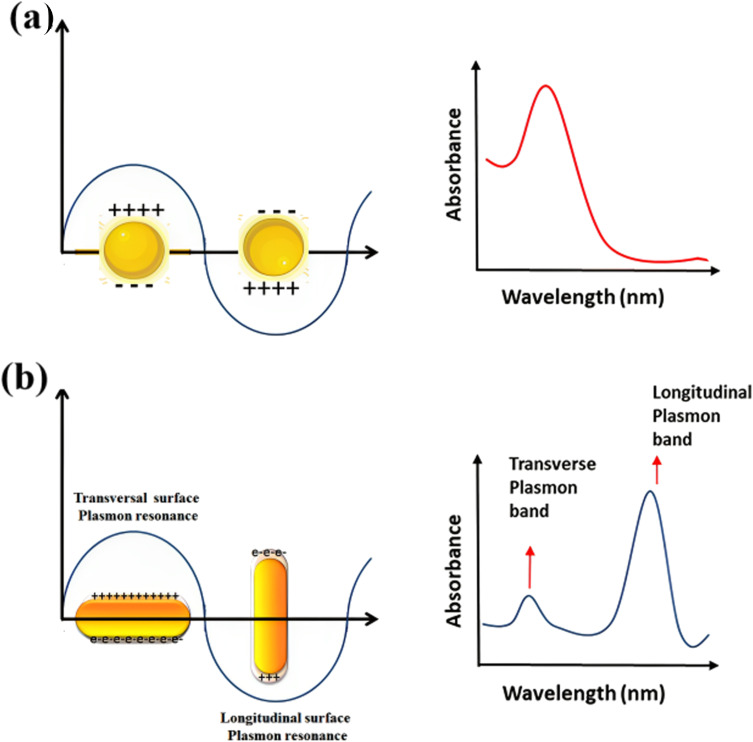
Schematic of plasmon oscillation and measured absorbance spectra of (a) G nanoparticles and (b) (GNR) display the transverse and longitudinal SPR modes.

Because of their form anisotropy NRs own several advantages. They are desirable candidates for numerous biomedical uses because of *Tunable optical properties*: the aspect ratio of NRs may be precisely controlled during synthesis, allowing for tuning their optical properties. This means that the absorption and scattering of light may be adjusted to related wavelengths, including those in the visible and (NIR) regions. This tunability is considerable for imaging, photothermal therapy, and sensing uses. (i) NRs possess a larger surface area than their volume. This high surface-to-volume ratio enables efficient interactions with biological molecules, like drugs or targeting ligands for drug delivery and targeted therapy. The increased surface area also facilitates enhanced surface reactions and can improve the sensitivity of sensing platforms. (ii) The longitudinal aspect of NRs exhibits a strong LSPR in the NIR region, where biological tissues own low absorption and scattering. This makes NRs suitable for deep-tissue imaging and photothermal therapy. The LSPR impact can convert absorbed light energy into heat, leading to localized hyperthermia for cancer treatment or targeted tissue thermal ablation. (iii) The elongated shape of NRs can promote enhanced cellular uptake compared to spherical nanoparticles. This may be advantageous for targeted drug delivery and intracellular imaging, as the NRs can better penetrate cell membranes and access related cellular compartments. (iv) NRs typically exhibit greater mechanical stability than other nanostructures, like nanowires or nanotubes. This stability is paramount for their use in various biomedical uses, where the NRs need to withstand physiological conditions like shear forces and enzymatic degradation. (v) The NRs surfaces may be easily functionalized with various biomolecules, like antibodies, peptides, or aptamers, to enable targeted delivery, related recognition, or enhanced biocompatibility. Functionalization strategies allow for tailoring the NRs to related uses and can enhance their stability and biocompatibility in physiological environments.^74–77^ NRs are made of (G) seeds anisotropically, and on their sides, crystal plains (110) and (100) are created.^78–80^ Compared to the (100) and (110), the facet (111) has the shortest Au-(G)atomic gap (110).^81,82^ The big trimethyl ammonium head group is easily preferred for binding along the loosely packed facets (100) or (110).^83^ As a result, the surfactant molecules form bilayers and preferentially adsorb on the side facets. For instance, elemental (G) is created when CTA^+^Au(i) and (AA) react on the GNP surface.^84–86^ These reagents must first transform from bulk liquid to the bilayer's seed surface for spherical nanoparticles. Then, they must dissolve in the bilayer matrix before interacting on the nanoparticle's surface. In contrast, there are only two processes in the case of NRs: the diffusion of bulk liquid to the NR tip and the reaction on the surface of the tip of NRs. The NP grows when the generated elemental (G) is deposited on the already-existing particle surface. The big trimethyl ammonium head group is easily preferred for binding along the loosely packed facets (100) or (110).^83^ Preferentially, the surfactant molecules adsorb on the side facets and form bilayers.^87–89^ The (G) synthesis with well-defined sizes and shapes has received much interest because of its significance in the electrical and optical features of these NRs. Changing the longitudinal Plasmon absorption bands of (GNR) and adding NIR absorption ba NRs at the necessary wavelength is possible by changing the aspect ratio.^83,90–92^ Even a small change in aspect ratio results in a noticeable shift in the NIR absorption wavelength.^93–97^ (SPR) bands are two absorption bands that (GNR) exhibits, one of which is TSPR (transverse) in the visible spectrum, and the other is LSPR (longitudinal) in the near-infrared spectrum (NIR). Fig. 3 exhibits two distinct plasmon oscillation modes of (GNR), the transverse and longitudinal (SPR). The transverse SPR mode corresponds NRs to the collective oscillation of conduction electrons perpendicular to the long axis of the NDs. In contrast, the longitudinal SPR mode refers to oscillating along the ND's long axis. The schematic representation would display the NR structure with arrows indicating the direction of electron oscillation for both the transverse and longitudinal modes. The absorbance spectra of (GNR) solution are typically measured using techniques like UV-vis spectroscopy. The spectra display the absorption intensity as a function of wavelength. For (GNR), the spectrum typically exhibits two distinct peaks corresponding to the transverse and longitudinal SPR modes. The transverse SPR peak is observed at shorter wavelengths in the visible region, while the longitudinal SPR peak appears at longer wavelengths in the (NIR) region. The exact positions of these peaks depend on the size, aspect ratio, and local environment of the (GNR). These measured absorbance spectra provide valuable inproduction about the optical feature of (GNR), including the wavelengths at which they strongly interact with light. This knowledge is essential for various uses, like designing NRs for related imaging or therapeutic purposes and optimizing their performance in biomedical and nanophotonic systems.

Table 2 summarizes the controlling factors for the morphology of gold nanoparticles, including nanorods, nanowires, nanobelts, and nanoparticles, involving precise manipulation of multiple factors. For nanorods, adjusting the aspect ratio through growth agent ratios, surfactant concentrations, reaction conditions, and seed particle shapes dictates their elongated form. Nanowires' diameters depend on catalyst sizes, growth temperatures, catalyst types, and precursor materials. Nanobelts, composed of various materials, are influenced by precursor choice, growth conditions, catalysts, and crystallographic orientations. Nanoparticles exhibit size and shape variations due to nucleation and growth kinetics, reaction temperatures, reducing agent selection, and stabilizing agents. These factors demonstrate the intricate interplay in achieving desired gold particle morphologies for diverse nanotechnology and materials science applications.

**Table 2:** The factors that affect the morphology of gold particles

	Factors affecting morphology	Details and considerations
Nanorods	(1) Aspect ratio (length-to-diameter ratio)	Controlled during synthesis by adjusting the ratio of growth agents, such as silver ions to gold ions
	(2) Presence of surfactants	Surfactants like CTAB (cetyltrimethylammonium bromide) direct growth along specific facets, leading to rod-like shapes
	(3) Reaction conditions	Temperature, pH, and concentration of reactants impact the growth rate and final aspect ratio
	(4) Seed particle shape	The shape of seed particles acts as a template, influencing the final rod shape
Nanowires	(1) Diameter control	Diameter can be controlled by the size of the catalyst nanoparticles or growth conditions
	(2) Growth temperature	Higher temperatures can lead to faster growth and larger diameters
	(3) Catalyst type	Different catalyst materials initiate and guide wire growth differently
	(4) Precursor material	The choice of gold precursor can affect wire formation and composition
Nanobelts	(1) Precursor material	The use of different materials for the precursor can lead to varied belt compositions, such as metal oxides or carbon
	(2) Growth conditions	Factors like temperature, pressure, and reactant concentration influence the morphology of nanobelts
	(3) Catalysts	Catalysts can be employed to initiate and guide the growth of nanobelts in desired directions
	(4) Crystallographic orientation	The orientation of crystallographic planes impacts the width and length of nanobelts
Nanoparticles	(1) Nucleation and growth kinetics	The nucleation and growth rate affect particle size and shape, with slower growth leading to larger particles
	(2) Reaction temperature	Higher temperatures can promote rapid nucleation and growth, leading to smaller nanoparticles
	(3) Choice of reducing agent	The reducing agent is critical in reducing gold ions to form nanoparticles, influencing size and shape
	(4) Stabilizing agents	Surfactants or capping agents are used to stabilize nanoparticles and can impact their final shape and dispersibility

### 2.1 Electrochemical preparation of (GNR): a pathway for controlled synthesis and tailored optical features

The electrochemical preparation method of (GNR) involves the electrodeposition of (G) onto a conductive substrate in the existence of a surfactant or template that controls the growth of (NRs). This method offers a synthetic pathway for producing (GNR) in large yields and allows for precise control over their size, aspect ratio, and optical features. Here is a general overview of the electrochemical preparation method for (GNR):

(1) *Conductive substrate*: a conductive substrate, like a glassy carbon or (G)-coated electrode, is prepared and thoroughly cleaned to ensure a clean and stable surface for electrodeposition.

(2) *Electrolyte solution*: an electrolyte solution typically contains a (G) precursor salt, a supporting electrolyte, and a surfactant or template molecule. The (G) precursor salt is commonly a (G) chloride compound (*e.g.*, HAuCl_4_), while the supporting electrolyte is often a salt like KCl or NaCl.

(3) *Electrochemical cell setup*: the cleaned conductive substrate is placed as the working electrode in an electrochemical cell. A counter electrode (*e.g.*, platinum wire) and a reference electrode (*e.g.*, Ag/AgCl electrode) are also included in the cell.

(4) *Electrodeposition process*: the electrodeposition is carried out by applying a controlled potential or current to the working electrode while stirring the electrolyte solution. The potential or current is adjusted based on the desired growth conditions for NRs. The surfactant or template molecule in the electrolyte solution plays a considerable role in controlling the anisotropic NRs growth.

(5) *Growth and production of NRs*: under the applied potential or current, (G) ions from the electrolyte solution are reduced and deposited onto the working electrode surface. The surfactant or template molecule directs the NRs growth by select binding to certain crystal facets of (G), favoring elongated growth along related directions. This results in the production of (GNR) with controlled dimensions and aspect ratios.

**Fig. 4:**
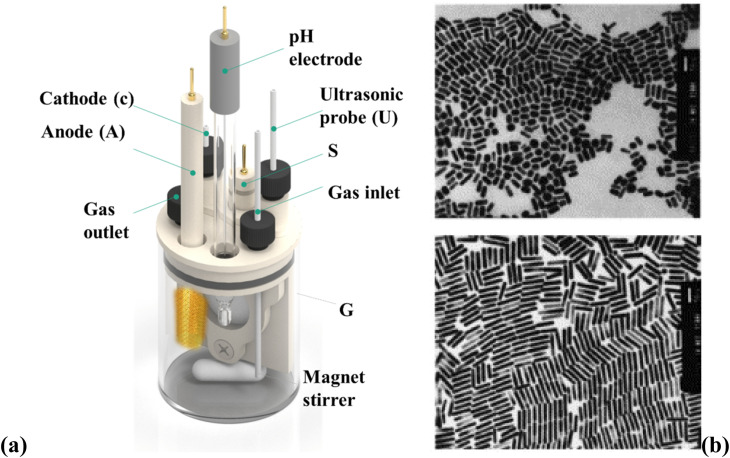
(a) Shows a schematic diagram of the setup for (GNR) electrochemical reparation and (b) shows (TEM) images of (GNR) with various aspect ratios. They are reprinted with permission from ref. 100 © 1999 American Chemical Society.

The electrochemical method offers control over the (GNR) growth and allows for synthesizing tailored NR structures. By adjusting the experimental conditions, like the applied potential, electrolyte composition, and surfactant/template molecules, the size, aspect ratio, and optical features of the (GNR) may be precisely controlled. This versatility makes the electrochemical preparation method suitable for various nanotechnology, sensing, and biomedicine uses. The electrochemical preparation method of (GNR) involves the electrodeposition of (G) onto a conductive substrate in the existence of a surfactant or template that controls the NRs growth is general in Fig. 4a. Fig. 4a shows a schematic diagram of the setup for (GNR) electrochemical reparation. The setup components include VA: power supply, which provides the necessary voltage or current for the electrochemical process. G: glassware electrochemical cell, where (GNR) electrodeposition occurs. It is a container that holds the electrolyte solution and the electrodes. T: Teflon spacer acts as a separator among the electrodes to prevent direct contact. S: electrode holder, which holds the working electrode (substrate) in place during the electrochemical process. U: ultrasonic cleaner used for cleaning the electrodes or other components before the experiment. A: anode, the electrode where oxidation occurs during the electrochemical process. C: cathode, the electrode where reduction occurs during the electrochemical process. Fig. 4b shows (TEM) images of (GNR) with various aspect ratios. The aspect ratio is the length to the width of the NRs, indicating their elongated shape. The top image represents (GNR) with an aspect ratio of 2.7, while the bottom image shows (GNR) with an aspect ratio of 6.1. The scale bars in the images indicate a length of 50 nm. Pérez-Juste *et al.*^98^ investigate the influence of various parameters, including electric field strength, surfactant concentration, and pH, on the production and (GNR) growth. The study provides valuable insights into the mechanism behind (GNR) growth and offers a novel approach to achieving desired morphologies. By manipulating the electric field, the researchers control the growth process, producing well-defined NRs. In this example, a (CTAB) surfactant is utilized to direct the (GNR) growth during the electrodeposition process. The CTAB surfactant binds select to the <100> crystal facets of (G), promoting the elongation of NRs along the <100> direction. By manipulating the concentration of the CTAB surfactant in the electrolyte solution, researchers can control the aspect ratio of the resulting (GNR).^99^ The electrochemical cell setup includes a glassy carbon electrode as the working electrode, a platinum wire as the counter electrode, and an Ag/AgCl electrode as the reference electrode. The electrolyte solution contains a (G) precursor salt, like HAuCl_4_, a supporting electrolyte (*e.g.*, KCl), and the CTAB surfactant. A controlled potential or current is applied to the working electrode during electrodeposition, while the electrolyte solution is stirred. Under the applied potential or current, (G) ions are reduced and deposited onto the working electrode surface, guided by the CTAB surfactant. The selective binding of the surfactant to related crystal facets promotes (GNR) growth with controlled dimensions and aspect ratios.

The resulting (GNR) exhibits tailored optical features because of their related aspect ratio. The longitudinal plasmon resonance of (GNR), which determines their absorption and scattering properties, may be finely tuned by adjusting the aspect ratio. For example, increasing the aspect ratio results in a redshift in the plasmon resonance, enhancing optical features in the near-infrared region. This is particularly valuable for photothermal therapy and biomedical imaging, where efficient light absorption and localized heat generation are desired.

The electrochemical preparation of (GNR) involves multiple steps and factors influencing their production and morphology. This process includes the dissolution of a (G) anode, the migration of (G) anions to the cathode, the reduction of (G) ions, and the role of cationic surfactants in the complex formation. The exact location of nucleation, whether inside micelles or on the cathode surface, is still not fully understood and requires further investigation. Sonication is typically employed to detach the formed NRs from the cathode surface or to shear them as they grow. The existence of an Ag (silver) plate in the electrolytic solution has a noticeable impact on the aspect ratio of the (GNR). The redox reaction among (G) ions from the anode and the Ag metal produces Ag ions, which play a role in determining the length of the nanorods. The exact mechanism and function of Ag ions in this process are not entirely elucidated. It is speculated that the Ag layer formed on the longitudinal faces of (GNR) inhibits their growth in the transverse direction, contributing to aspect ratio control. One advantage of the electrochemical approach is its ability to yield high targets (GNR). However, a drawback is that the process may be time-consuming. Further research is necessary to fully understand the intricacies of the electrochemical preparation of (GNR), including the precise role of Ag ions and the optimization of process parameters to enhance efficiency and control over the nanorod features.^101^

### 2.2 Template method: controlled synthesis of (GNR) for optics and biomedical uses

The template method is a well-established technique that provides excellent control over the size, aspect ratio, and optical features of (GNR) by adjusting various synthesis parameters. The method relies on the electrochemical deposition of (G) inside nanoporous polycarbonate or alumina template membranes, enabling precise control over the resulting nanorod morphology. Here is an overview of the template method:

(1) *Template selection*: nanoporous polycarbonate or alumina membranes are chosen as templates. These membranes own well-defined nanopores that serve as a framework for nanorod growth. A thin layer of silver (Ag) or copper is sputtered onto the template membrane to provide a conductive base for electrodeposition.^102,103^

(2) *Electrochemical growth*: (GNPs) are electrochemically grown on the conductive layer. Subsequently, (G) is electrodeposited into the nanopores of the template membrane. The length of the resulting NRs is controlled by the amount of (G) deposited, while the diameter of the (GNPs) matches the pore diameter of the template membrane.

(3) *Removal of layers*: the copper or silver layer and the template membrane are removed, leaving behind the (GNR). To stabilize the NRs and prevent aggregation, a polymeric material like poly(vinyl pyrrolidone) is commonly used.^104^

The template method directs the (GNR) growth, resulting in (GNR) with precise sizes and shapes. Liao *et al.*^105^ employed the template method to fabricate ordered arrays of (GNR) with excellent reproducibility for enhanced Raman spectroscopy uses. In the study, the authors aimed to overcome the limitations of conventional SERS substrates, which often suffer from poor reproducibility and difficulty achieving uniform enhancement. They developed a template-based approach to fabricate well-ordered arrays of (GNR) with controlled dimensions and spatial distribution. The authors used anodization techniques to create nanoporous AAO templates with a regular hexagonal array of pores. The pore size was carefully controlled to achieve the desired dimensions of the (GNR). The AAO templates were then subjected to an electrochemical deposition process to grow (GNR) within the nanopores. The deposition process involved the application of a potential difference among the AAO template and a counter electrode in a (G) precursor solution. This resulted in the controlled (GNR) growth within the pores. After the (GNRs) were formed, the AAO template was dissolved, leaving behind the (GNR) arrays. The (GNR) arrays were then transferred onto suitable substrates for subsequent characterization and SERS measurements.

The fabricated (GNR) arrays exhibited excellent reproducibility regarding their size, shape, and distribution. The well-defined geometry and regular arrangement of the NRs contributed to uniform SERS enhancement across the substrate surface. The researchers demonstrated the enhanced SERS performance of their (GNR) arrays by measuring the Raman signals of various analytes. This study highlights the template-based approach as a promising method for the controlled synthesis of (GNR) arrays with high reproducibility, providing a platform for efficient SERS uses. The well-defined displays offer enhanced sensitivity and uniformity, making them suitable for various sensing and spectroscopic uses that rely on SERS.

Sornsanit *et al.*^106^ aimed to enhance the antibacterial features of ZnO NRs by decorating them with AuNPs. ZnO is known for its antimicrobial properties, while AuNPs exhibit excellent biocompatibility and antibacterial impact. By combining these two materials, the researchers aimed to create a synergistic effect that could enhance the antibacterial activity against harmful microorganisms. First, The ZnO NRs were synthesized using a hydrothermal method. The reaction involved the growth of ZnO NRs on a substrate by controlling the reaction conditions, like temperature and precursor concentration. The resulting ZnO NRs had a well-defined morphology and crystalline structure. The ZnO NRs were then decorated with AuNPs through a deposition process. The AuNPs were synthesized separately and then deposited onto the ZnO nanorods surface. The deposition was achieved through chemical bonding and van der Waals forces. The synthesized ZnO NRs decorated with AuNPs were characterized using scanning electron microscopy (SEM) and X-ray diffraction (XRD) to analyze their morphology and structure. The antibacterial activity of the nanocomposite was evaluated by conducting antibacterial tests against related bacteria strains. The study results showed that the ZnO NRs decorated with AuNPs exhibited enhanced antibacterial activity compared to the pure ZnO nanorods. AuNPs on the ZnO surface increased the contact area with bacteria, leading to improved antibacterial efficiency. The researchers observed a noticeable reduction in bacterial growth when exposed to the nanocomposite material. The seed-mediated growth method is a widely employed for synthesizing gold nanorods (GNRs) with precise control over their size and aspect ratio. The process begins by preparing small gold nanoparticles (seeds) typically around 5–10 nanometers in size. These seeds act as nucleation sites for GNR growth. The size and shape of the seeds can be controlled by adjusting the ratio of gold precursor to reducing agent and the type of surfactant used. A growth solution is created by mixing a gold precursor (commonly chloroauric acid, HAuCl_4_) with a cationic surfactant such as cetyltrimethylammonium bromide (CTAB). The surfactant stabilizes the growing GNRs and helps control their shape. The growth solution is then introduced to the seed solution. Adding a mild reducing agent, typically ascorbic acid or another reducing agent, initiates the reduction of gold ions in the growth solution onto the seed surfaces. Notably, the CTAB surfactant directs the growth of anisotropic nanorods instead of spherical nanoparticles. By adjusting the concentration of CTAB and the growth time, it is possible to finely control the aspect ratio (length-to-width ratio) of the resulting GNRs. Longer growth times or higher CTAB concentrations tend to produce longer GNRs. After synthesis, GNRs may need to be purified to remove excess surfactants and byproducts. This is typically done through centrifugation and redispersion. The synthesized GNRs should be characterized using TEM and UV-vis spectroscopy to confirm their size, shape, and optical properties, including the characteristic longitudinal and transverse surface plasmon resonance (SPR) peaks. The seed-mediated growth method provides precise control over GNR dimensions, making it a favored choice for various applications such as biomedical imaging, drug delivery, and photothermal therapy. Researchers can fine-tune the method to produce GNRs with specific optical properties and aspect ratios to suit their needs.

The template method offers the flexibility to produce (GNRs) of various sizes by modifying the pore diameter of the template membrane. However, it is paramount to note that the yield of NRs in this method is low, making it challenging to produce large quantities. Despite this limitation, the template method has played a considerable role in demonstrating several fundamental optical impacts and has found numerous uses in research and biomedical fields where precise control over nanorod morphology is required.

### 2.3 Photochemical preparation method of (GNR)

The synthesis of (GNR) using the photochemical method offers a versatile approach to controlling their size and aspect ratio. This method involves utilizing photochemical reactions to reduce (G) ions (Au^3+^) and shape them into NRs (NRs). The synthesis process typically includes the following steps:

(1) *Reduction of (G) salts*: chloroauric acid (AuCl_4_^−^) solutions are prepared as a source of (G) ions (Au^3+^). A reducing agent, like (AA) (AA), is added to convert Au^3+^ ions into monovalent (G) ions (Au^+^).

(2) *UV irradiation*: the mixture of Au^+^ ions and the reducing agent solution is exposed to ultraviolet (UV) light. The UV light acts as an additional reducing agent, converting Au^+^ ions into metallic (GNPs) (Au^0^).

(3) *Shape control*: various factors, like irradiation time, the existence of surfactants, and manipulation of silver ions (Ag^+^), are employed to control the growth and shape of the nanorods. Anisotropic growth, leading to elongated rod-like structures, may be achieved by adjusting the duration of UV irradiation.

The photochemical method allows for precise control over the synthesis process, enabling the production of (GNR) with desired anisotropic shapes and optical properties. By adjusting parameters like UV irradiation time, surfactant concentration, and the existence of other ions, researchers can tailor the size and aspect ratio of the synthesized nanorods. The method involves the reduction of (G) ions and their subsequent shaping into NRs through photochemical reactions.

Niidome *et al.*^107,108^ utilized a photochemical method for synthesizing (GNR). The process involved the reduction of (G) salts (chloroauric acid solutions) with (AA) (AA) and exposure to varying levels of ultraviolet (UV) irradiation. The reduction of (G) salts transfers Au^3+^ ions into monovalent Au^+^ ions, and further UV irradiation acts as a reducing agent to convert Au^+^ ions into metallic (GNPs) (Au^0^). The addition of acetone played a role in promoting the anisotropic (GNR) growth, which varied in aspect ratio and shape depending on the duration of UV irradiation. This chemical method controlled GNR growth and dimensions by adjusting the UV irradiation time.

In contrast, Placido *et al.*^109^ employed a photochemical method involving a double-surfactant solution and UV irradiation. The solution contained two surfactants, tetradecyl amine (TC12AB) and (CTAB), which stabilized the (GNPs) (GNPs) and controlled the NRs growth (NRs). Chloroauric acid (HAuCl_4_) served as the source of (Au^3+^) ions, and UV irradiation at a wavelength of 254 nm acted as a reducing agent, converting Au^3+^ ions into metallic (GNPs). Adjusting the concentration of Ag^+^ ions in the reaction mixture allowed for control over the growth and shape of the NRs, while the UV light intensity and irradiation time influenced the reduction of (G) ions and the subsequent NRs growth. Precise optimization of these parameters was considerable for obtaining consistent and desired results in synthesizing (GNR) using the photochemical method.

It is paramount to note that variations in parameters like Ag^+^ ion concentration, UV light intensity, and irradiation time can introduce fluctuations in the quality of the synthesized (GNR). Therefore, precise control and optimization of these parameters are necessary to achieve consistent and desired results in GNR synthesis using the photochemical method.

**Fig. 5:**
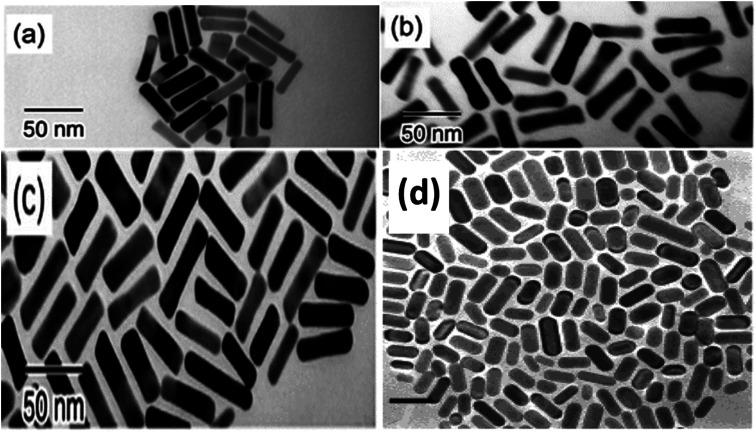
The TEM images display the (GNR) synthesized by UV irradiation for various durations. Each image is labeled with its corresponding irradiation time: (a) for 5 minutes, (b) for 30 minutes, and (c) for 50 minutes. This analysis provides visual evidence of how the aspect ratio and shape of the (GNR) vary with various UV irradiation times^107^ © The Royal Society of Chemistry 2003 (d),^110^ Copyright © 2002 American Chemical Society.

Fig. 5 illustrates the obtained (GNR) with various aspect ratios (AA) from manipulating Ag content and the photochemical synthesis process.

The description highlights the critical steps involved in each method, including reducing (G) salts, reducing agents' role, using surfactants, and the influence of parameters like UV irradiation, Ag ion concentration, and reaction time.

The template method uses a template like (GNR) to guide the growth of new nanorods. The process involves depositing a conductive layer on the template membrane, electrochemically growing (GNPs) on the conductive layer and subsequently depositing (G) into the nanopores of the template membrane. The template method controls the size, aspect ratio, and optical features of the resulting (GNR).

On the other hand, the photochemical method relies on photochemical reactions to reduce (G) ions and shape them into nanorods. Various variations of the photochemical process are described, involving the use of reducing agents like (AA), the existence of surfactants like CTAB and TC12AB, manipulation of Ag ion concentration, UV irradiation, and precise control over parameters like UV light intensity and irradiation time. These variations enable the controlled synthesis of (GNR) with desired aspect ratios and characteristics.

It is paramount to note that both methods offer advantages and challenges. The template method provides reasonable control over the size and aspect ratio but may have limitations regarding yield. The photochemical process allows for precise tuning of GNR characteristics but requires careful parameter optimization to ensure consistent results.

### 2.4 Seed-mediated growth method of prepared (GNR)

**Fig. 6:**
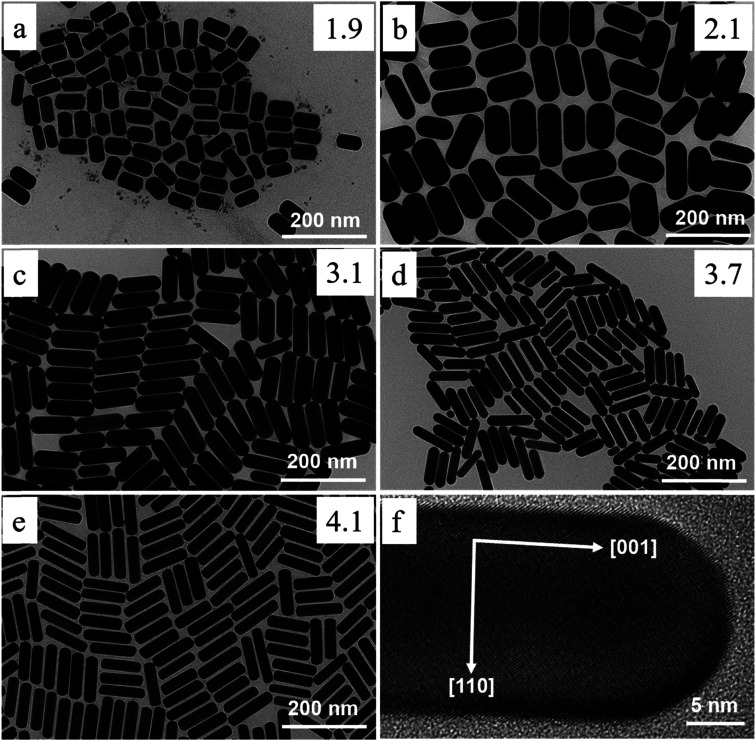
Depicts synthesized (GNR) TEM images with variations in CTAB concentration and AgNO_3_ concentration. In images (a) to (e), the CTAB concentration was varied, with (a) at 0.008 M and (b–e) at 0.01 M. Each image corresponds to various concentrations of AgNO_3_: (a) 0.144 mM, (b) 0.032 mM, (c) 0.144 mM, (d) 0.240 mM, and (e) 0.048 mM. The concentration of NaOL used throughout the synthesis process was 0.005 M, and the volume of HCl added was 0.3 mL. Image (f) shows a TEM image of the (GNR) from the same sample as in image (e). The (GNR) exhibits a rod-shaped morphology, with the crystallographic 〈001〉 direction along the long axis of the rod, as indicated by the white arrows in the image (f), reproduced with permission from ref. 113 © 2021 American Chemical Society.

Recent research has successfully controlled the (GNR) distribution in the range of 5–40 nm, and the size may be changed by adjusting the ratio of seed and metal salts.^104,111^ Controlling various variables in the seed-mediated growth method for (GNR) is highlighted here. (i) *Reducing agents*: the choice of reducing agent is considerable for preventing the nucleation of (GNR). In this case, a reducing agent called sodium borohydride (NaBH_4_) is used. After rapid stirring, it is added to the seed solution, forming a brown-yellow solution. (ii) *Seed metal salt solutions*: the seed solution consists of a (G) metal salt, in this case, HAuCl_4_, mixed with a surfactant solution. The related concentration of HAuCl_4_ and surfactant (C16TAB) is mentioned in the passage (2.0 mL of 5 × 10^−4^ M HAuCl_4_ with 5.0 mL of 0.20 M C16TAB). (iii) *Capping agent*: the surfactant solution acts as a capping agent, providing stability and controlling the growth of (GNR). In this case, C16TAB is used as the surfactant, and its concentration is adjusted accordingly. (iv) *Temperature and stirring*: the (G)seed solution is held at 25 °C without stirring after adding the reducing agent. By carefully controlling these variables, achieving the desired production of (GNR) with the desired morphology, size, and aspect ratio is possible. The seed-mediated growth method allows fine-tuning these parameters to obtain (GNR) with related application features.^112^ By carefully adjusting the concentration of AgNO_3_ and other growth conditions, researchers can achieve desired outcomes regarding yield, aspect ratio, crystal structure, and optical features of (GNR).^104^ Wei *et al.*^113^ used low concentrations of CTAB (0.008 M) in the growth solution, Wei *et al.* successfully synthesized (GNR) with variable aspect ratios. The related aspect ratios achieved were not mentioned in the provided information. However, by modifying the amounts of NaOL, AgNO_3_, HCl, and seeds, the growth conditions may be adjusted to control the aspect ratio of the (GNR), as described in the previous response. In addition, Wei and their coworkers^114^ optimized the conditions of (GNR) growth by managing the AgNO_3_ amount and/or HCl concentration. The seed solution was prepared by mixing 0.25 mL of HAuCl_4_ at a concentration of 10 mM with 10 mL of CTAB solution at a concentration of 0.1 M. The mixture was vigorously stirred, and then a reducing agent, 0.6 mL of 10 mM NaBH_4_, was added. The resulting solution changed color from yellow to brownish yellow. The seed solution was stored at a temperature of 30 °C for 30 minutes. In a vial, 2.5 mL of CTAB at a concentration of 0.1 M was added to create the growth solution. Then, 0.037 g of sodium oleate (NaOL) was dissolved in 21.25 mL of bidistilled H_2_O at a temperature of 45–50 °C. Subsequently, a solution of 4 mM AgNO_3_ (0.9 mL) was added to the vial once the temperature of the solution reached 30 °C. After 15 minutes, 0.25 mL of 10 mM HAuCl_4_ was added to the mixture. The solution was stirred for 90 minutes, during which the color of the solution changed. The pH of the solution was made acidic by adding 0.3 mL of 37% HCl. After 15 minutes, the seed solution (0.04 mL) and 0.075 mL of 64 mM (AA) (AA) were added to the mixture. The solution was allowed to develop undisturbed at 30 °C for 12 hours. During this time, the GNDs formed. Fig. 6a–e shows the TEM images of synthesized (GNR) at low concentrations of CTAB (ranging from 0.008 to 0.010 M) were observed. The aspect ratio (AR) of the (GNR) varied from 1.9 to 4.1. The related details about NaOL, AgNO_3_, and HCl amounts are worth noting.

**Fig. 7:**
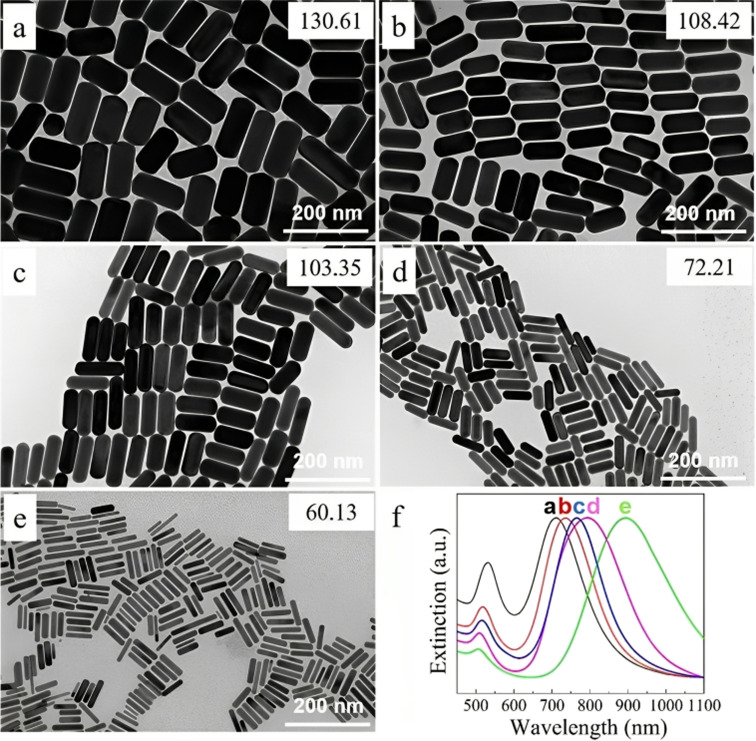
(GNR) synthesized by various seed solution amounts of 0.005, 0.01, 0.02, 0.04, and 0.16 mL in TEM images from (a to e). (f) Normalized extinction spectra of the (GNR) are shown in (a)–(e), reproduced with permission from ref. 113 © 2021 American Chemical Society.

Also, the amount of seed solution is paramount in the size change, as shown in Fig. 7. The length and diameter of the (GNR) rose when the seed particle amount was decreased. The (G)precursors/seeds ratio rises when fewer seeds are in the growing solution. Thicker and larger (GNR) form because each seed binding site receives more (G) precursors. Ye, Xingchen, *et al.*^115^ showed that utilizing binary surfactants in synthesizing (GNR) allows for creating thicker NRs by using a smaller amount of seed in the final growth medium. Fig. 7a–e depict the corresponding (GNR) with various aspect ratios. As more seeds are added to the growth medium, the aspect ratio of the NRs increases, ranging from 2.1 to 4.9. This aspect ratio variation is shown in Fig. 7a–e. The corresponding extinction spectra of these GNDs are presented in Fig. 7f. The spectra display the absorption peaks related to the (LSPR) of the NRs. As the aspect ratio increases, the LSPR peak undergoes a redshift, shifting from 709 nm to 896 nm. This redshift in the LSPR peak results from the increasing aspect ratio of the (GNR).

#### 2.4.1 Preparation of (GNR) in percent of AgNO_3_

Preparing (GNR) involves controlling various parameters to achieve the desired properties, including the aspect ratio, size, and optical features. One paramount factor in the synthesis process is the concentration of AgNO_3_, which plays a considerable role in determining the final characteristics of the (GNR). The concentration of AgNO_3_ is typically adjusted to influence the growth and shape of the (GNR). By modifying the amount of AgNO_3_ in the growth solution, researchers can achieve related outcomes regarding yield, aspect ratio, crystal structure, and optical features of the (GNR). The corresponding concentration of AgNO_3_ used to prepare (GNR) can vary depending on the desired results and the experimental conditions. Researchers often optimize the concentration of AgNO_3_ to obtain the desired aspect ratio and control the (GNR) growth impact. It is worth noting that the concentration of AgNO_3_ should be carefully selected, as it can own a noticeable impact on the synthesis process. Higher concentrations of AgNO_3_ may result in faster growth rates and longer (GNR), while lower concentrations can result in shorter (GNR) with a lower aspect ratio. The choice of AgNO_3_ concentration is typically made based on the related requirements of the intended application of the (GNR). Various concentrations can lead to variations in the optical properties, like the plasmonic resonance wavelength, which is paramount for sensing, imaging, and photothermal therapy.

For example, in a growth solution with a total volume of 10 mL, the composition could be as follows: (G)precursor (*e.g.*, HAuCl_4_): 0.25 mL of a 10 mM solution; surfactant (*e.g.*, CTAB): 2.5 mL of a 0.1 M solution; Reducing agent (*e.g.*, (AA)): 0.075 mL of a 64 mM solution; hydrochloric acid (HCl): 0.3 mL of 37% (v/v); and AgNO_3_ solution: 0.2 mL (2% of 10 mL).

Adding AgNO_3_ during the synthesis of (GNR) is essential as it enhances the yield and controls the aspect ratio and shape of the nanorods. AgNO_3_ acts as a shape-directing agent, playing a considerable role in producing (GNR). When AgNO_3_ is introduced into the synthesis process, it undergoes a redox reaction with the (G) ions generated by the anode. This reaction results in the release of Ag ions (Ag^+^) into the solution.

The Ag ions prefer to adsorb onto related crystallographic facets of the growing (GNR), influencing their growth direction and shape. By select adsorbing onto these facets, the existence of Ag ions helps inhibit the growth of certain crystallographic facets and promotes the elongation of the NRs along their longitudinal axis. This mechanism contributes to a higher aspect ratio of (G).

When Ag nitrate is introduced into the solution, the Ag ions (Ag^+^) interact with the (G) ions (Au^3+^) present in the system. These Ag ions prefer to adsorb onto related crystallographic facets of the growing nanoparticles, which significantly influences the growth direction and shape of the particles. This selective adsorption of Ag ions onto certain facets plays a considerable role in determining the morphology of the NRs.^116–118^

It is worth noting that the pH of the solution impacts the reducing power of the common reducing agent used in GNR synthesis, ascorbate. At lower pH levels, like pH 2.8, in this case, the reduction of ascorbate is enhanced. Consequently, under these experimental conditions, the Ag ions (Ag^+^) are not likely to be directly reduced themselves, but rather their existence primarily affects the structure and growth of the (GNR).^119^ Furthermore, the concentration of Ag ions in the solution influences the growth kinetics and crystal structure of the (GNR). As the concentration of Ag ions increases, it directly affects the nanorods' aspect ratio and relative dimensions. This, in turn, results in a redshift of the longitudinal plasmon band of the nanorods, causing it to shift towards longer wavelengths in the UV-visible spectrum. The optical features of the (GNR) can thus be tuned by adjusting the concentration of Ag ions (137, 138). The concentration of AgNO_3_ in the growth solution plays a considerable role in fine-tuning the aspect ratio and dimensions of the (GNR). By adjusting the concentration of AgNO_3_, researchers can impact control the existence of Ag ions (Ag^+^) in the solution, which influences the shape of the growing NRs.^92,120^ The (AA) reduction of Ag nitrate (AgNO_3_) on the (GNR) surface in the existence of surfactants like cetyltrimethylammonium bromide (C16TAB) and either citrate or polyvinylpyrrolidone (PVP) has been established as a more straightforward and more direct method for controlling the shape and optical features of (GNR). In this method, Ag nitrate is a source of Ag ions (Ag^+^), which interact with the (GNR) surface. (AA), a reducing agent is introduced into the system, reducing the Ag ions to metallic silver (Ag). This reduction process primarily occurs preferentially on the (GNR) surface, leading to the deposition of Ag atoms. By controlling the concentration and reaction conditions of AgNO_3_, (AA), and surfactants, researchers can precisely modulate the shape and optical features of (GNR). The existence of surfactants like C16TAB, citrate, or PVP plays a considerable role in stabilizing the (GNR) and facilitating the reduction process. These surfactants help prevent aggregation or undesired (GNR) growth, ensuring the production of well-defined and controlled structures.

The (AA) reduction method offers advantages in terms of simplicity and directness compared to other synthetic approaches. It enables the controlled deposition of Ag atoms onto the (GNR) surface, leading to modifications in their shape and optical characteristics. This method provides researchers with a versatile tool for tailoring the features of (GNR) for various uses, including biomedical imaging, sensing, and drug delivery.^104,121–124^ Huang *et al.*^125,126^ the focus was synthesizing Au@Ag core–shell NRs (NRs) in an alkaline surfactant solution with controlled pH. The researchers employed (AA) as a reducing agent to reduce Ag nitrate (AgNO_3_) on the (GNR) surface in an aqueous surfactant medium. The pH of the solution played a considerable role in this synthesis process, and glycine buffers were used to control the pH in the growth solution. The pH of the solution and the concentration of Ag ions had a noticeable impact on the final shape of the particles. The existence of the surfactant cetyltrimethylammonium bromide (C16TAB) resulted in the observation of an anisotropic Ag coating on the (GNR). As the solution pH increased, the rate of Ag deposition also increased. This indicates that the pH of the solution influenced the reduction kinetics and the deposition of Ag atoms on the (GNR) surface. By controlling the pH and the concentration of Ag ions, the researchers could modulate the growth and deposition of Ag atoms, producing a well-defined Au@Ag core–shell structure on the (GNR). The anisotropic Ag coating provided additional functionality and optical features to the (GNR), which can benefit uses like plasmon-enhanced sensing or catalysis.

**Fig. 8:**
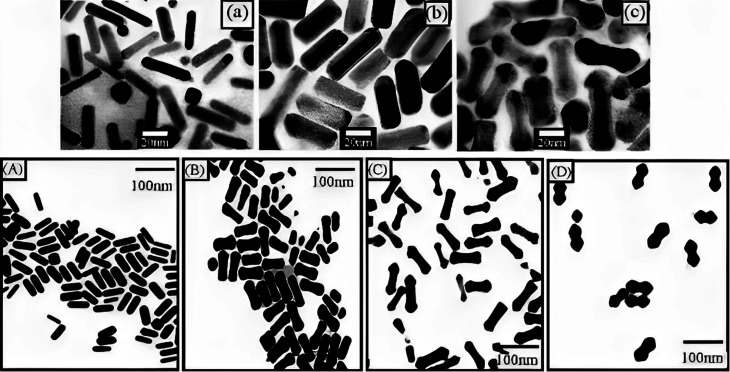
(a) TEM of (GNR), (b) Au@Ag with thin composite NRs, and (c) thick thin composite NRs with Ag layers. Permission from ref. 127 © 2001 American Chemical Society. Image bottom: (A) TEM of (GNR) as prepared, and (B) Au@Ag composite NRs prepared at the alkaline solution of surfactant at pH 8.0, (C) pH 9, and pH (D) 10 permission from ref. 125 © 2004 American Chemical Society.

The researchers observed an apparent dumbbell shape in the resulting particles, which was attributed to the faster deposition rate at the rods' tips compared to the rods' sides when the pH was higher (Fig. 8). Two possible explanations were proposed to account for these observations. Firstly, a C16TAB-driven variable rate of crystal production could result in a faster deposition rate at points of greater curvature, leading to the dumbbell shape. Alternatively, an electrochemical mechanism could be responsible, where regions of higher curvature experience a faster deposition rate. The related mechanism underlying these phenomena was not conclusion determined in the study. Huang *et al.* provided insights into the influence of pH and Ag concentration on the shape and growth of Au@Ag core–shell NRs, and proposed possible explanations for the dumbbell shape based on various crystal production rates or electrochemical impacts.

#### 2.4.2 Preparation of (GNR) without AgNO_3_

This method allows for synthesizing (GNR) without using AgNO_3_, providing a convenient and straightforward approach for obtaining well-defined NRs with controllable aspect ratios.

(I) Synthesis of 3.5 nm seed:

(1) A 20 mL solution of HAuCl_4_ (2.5 × 10^−4^ M) is mixed with a tri-sodium citrate solution (2.5 × 10^−4^ M) in a flask.

(2) While stirring, 0.6 mL of a cold 0.1 M NaBH_4_ solution is added to the mixture.

(3) The solution turns pink, indicating particle production. This solution is used as the seed solution.

(4) (TEM) is used to observe the particles with an average size of 3.5 ± 0.7 nm.

(II) Synthesis of 4.6 nm ± 1 aspect ratio rod:

(1) A growth solution containing 10 mL of HAuCl_4_ (2.5 × 10^−4^ M) and 0.1 M (CTAB) is prepared in a test tube.

(2) To the growth solution, 0.05 mL of a fresh solution of 0.1 M (AA) is added.

(3) Then, 0.025 mL of the 3.5 nm seed solution is added.

(4) The solution is not agitated or stirred, rods, spheres, and some plates with a 4.6 aspect ratio are present, which changes color to reddish-brown.

(5) The solution remains stable for over a month.

(III) Synthesis of 13 nm ± 2 aspect ratio rod:

(1) A three-step seeding procedure is followed. Three test tubes labeled A, B, and C are prepared, each containing 9 mL of growth solution.

(2) 0.05 mL of 0.1 M (AA), 2.5 × 10^−4^ M HAuCl_4_, and 0.1 M CTAB are added to each test tube.

(3) The 3.5 nm seed solution is added to test tube A (1.0 mL). After a few minutes, the color changes to red.

(4) After 4–5 hours, 1.0 mL of solution A is transferred to solution B and mixed. Solution B turns crimson.

(5) After another 4–5 hours, 1.0 mL of solution B is transferred to solution C. Within 10 minutes, solution C turns crimson.

(6) The solutions remain stable for over a month (GNR) with an aspect ratio of 13 in solution C.

(IV) Synthesis of 18 nm ± 2.5 aspect ratio rod:

(1) This process is similar to preparing 13 aspect ratio rods but with a variation in the order of adding the seeds.

(2) After the growth from the previous reaction is complete, seed solutions A and B are introduced to growth solution B for the 13 aspect ratio rods.

(3) While the particles in these solutions are still growing, particles from solutions A and B are transferred to the growth solution to create 18 aspect ratio rods.

(4) Centrifugation concentrates the long rods and separates them from the spheres and surfactant. The solid portion is then dispersed in water.

(5) The mechanism by which rod-shaped NPs develop in aqueous surfactant systems is not fully understood. Still, the preferred surfactant (C16TAB) adsorption to specific crystal faces is believed to control the growth process.^104,128,129^

The impact of various CnTAB analogs with varying hydrocarbon tail lengths on the synthesis of (GNR) was investigated. It was observed that the length of the surfactant tail played a considerable role in determining the size of the resulting NRs and their yield. Shorter chain lengths of the surfactant resulted in shorter (GNR) production, while longer chain lengths led to the synthesis of longer NRs. Moreover, longer surfactant chains were also found to contribute to larger yields of the desired NDs. The observed impact of surfactant tail length may be explained by considering the van der Waals interactions among the surfactant tails within the surfactant bilayer and on the (G)surface. It was proposed that a “zipping” mechanism takes place, where the preferential adsorption of C*n*TAB (with related hydrocarbon tail lengths) to various crystal faces occurs in a bilayer fashion. This zipping mechanism accounts for the selective growth of (GNR) with multiple aspect ratios, as the surfactant molecules adhere preferentially to related crystal faces of the increase in (GNR). The adsorption and growth rates on different crystal faces may be controlled by manipulating the surfactant tail length and synthesizing (GNR) with desired sizes and yields.^88^ The product of (GNR) synthesized using C16TAB-capped seeds is significantly higher compared to those without any surfactant (naked) or stabilized with citrate. This observation indicates that the colloidal stability of the (G)seed NPs plays a considerable role in enhancing the yield of NRs.^88,104^

**Fig. 9:**
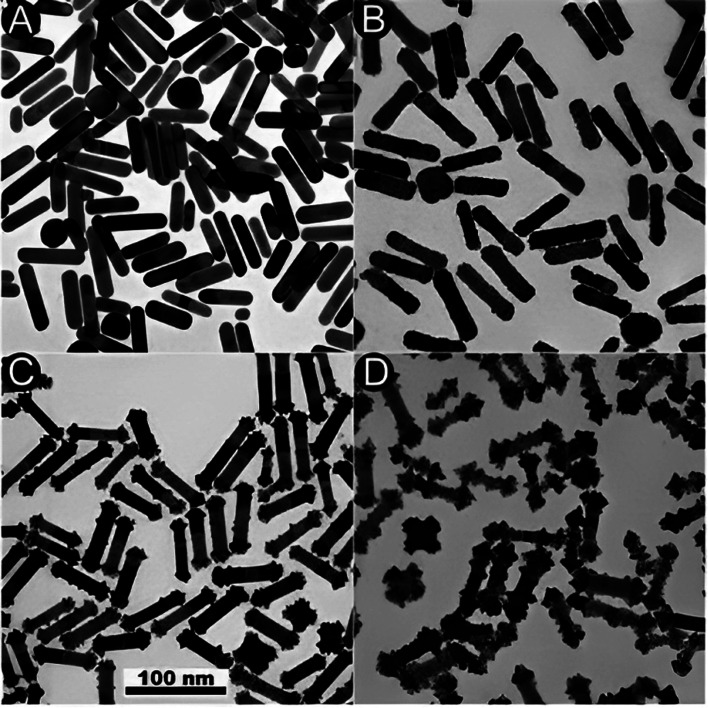
(A) and (B) TEM images of the initial (GNR) and Au@Pt formed in the absence or the existence (C and D) of Ag^+^, respect. The Pt : (G) molar ratios are 20% (C) and 100% (B, D). Reproduced with permission from ref. 124 © The Royal Society of Chemistry 2006.

Fig. 9 TEM images depicting the Pt reduction process with and without the existence of Ag^+^ ions. (A) TEM image showing the initial (GNR). (B) TEM image of (GNR) after reduction in the absence of Ag ions, resulting in homogeneous over-coating of the rods. (C and D) TEM images display the impact of Ag ions during the reduction process. In the existence of Ag ions, a dumbbell-like shape is observed, with platinum preferentially deposited on the tips of the rods. For low Pt concentrations, growth is rarely seen on the lateral sides of the rods. However, for higher Pt concentrations, unexpected change occurs, producing patches resembling pyramids on the lateral edges of the rods. Additionally, platinum deposition is observed on the tops of some (G) cubes. These observations suggest that Ag ions (Ag^+^) influence the reduction process and lead to related morphologies. The preferential deposition of platinum on the tips of the rods in the existence of Ag ions results in a dumbbell-like shape. The growth on the lateral sides of the rods is less pronounced at lower Pt concentrations but becomes more apparent at higher concentrations. This particular growth pattern indicates that the reduction rate is influenced by the frequency of micelle and NP collisions, similar to the production of (GNR) on these factors. In summary, adding Ag ions during the Pt reduction process affects the morphology of the resulting Au@Pt rods, with preferential deposition of platinum on the rod tips and pyramid-like patches on the lateral edges. These observations highlight the importance of controlling the reaction conditions and the existence of related ions in tailoring the morphology and composition of core–shell NRs (Table 3).

**Table 3:** Highlights the differences between (GNR) prepared with AgNO_3_ and (GNR) prepared without AgNO_3_

Aspect	GNRs prepared with AgNO_3_	GNRs prepared without AgNO_3_
Formation	Enhanced yield and control over aspect ratio and shape	Yield and shape control may be more challenging
Shape-control and directing agent	Enables control over the shape and aspect ratio of (GNR) through preferential adsorption of Ag ions onto related crystal facets, promoting elongation along the longitudinal axis	No related shape-directing agent is present, resulting in less precise control over the shape and aspect ratio of (GNR)
Crystallographic facets	Ag ions preferentially adsorb onto related crystal facets, promoting elongation along the longitudinal axis	Other factors or mechanisms may influence growth direction and shape
Aspect ratio	Maybe fine-tuned by adjusting the concentration of AgNO_3_	Aspect ratio control may be more limited
Yield	Improved yield by suppressing the production of unwanted shapes, like spherical nanoparticles	Yield may vary and may own a higher likelihood of unwanted shapes
Reduction process	Reduction of Ag ions to metallic silver occurs with (AA), preferentially on the surface of the nanorods	No related reduction process involving Ag ions
pH influence	Solution pH plays a considerable role, affecting the reducing power of (AA) and the Ag deposition rate. Higher pH and resulting Ag deposition rate increased	pH may still influence other aspects of the synthesis process
Core–shell structure	This can potentially result in the production of Au@Ag core–shell NRs	No related core–shell structure production involving Ag
Optical properties	Ag deposition and the involvement of Ag ions may cause a redshift in the longitudinal plasmon resonance band, shifting towards longer wavelengths in the UV-visible spectrum	Numerous optical and plasmon resonance phenomena exist due to the absence of Ag participation

It's important to note that the choice of the synthesis approach depends on various factors, including the desired nanorod characteristics, available resources, and the specific application. Researchers often weigh these advantages and disadvantages to select the most suitable method for their goals. Table 4 outline the advantages and disadvantages of various approaches for preparing gold nanorods.

**Table 4:** Summarized the advantages and disadvantages of various approaches for preparing GNR

Approach	Advantages	Disadvantages
Seed-mediated growth	Offers precise control over aspect ratio, enabling tailoring of optical properties	Involves a multistep synthesis process, potentially increasing complexity and time consumption
	Provides the ability to fine-tune the synthesis for monodisperse nanorods	Typically employs toxic surfactants like CTAB, posing safety concerns
	Suitable for both small- and large-scale production	Quality of seed particles can significantly impact the quality of final nanorods
	Enables the production of high-quality, uniform nanorods with high reproducibility	Scalability for large-scale production may be limited
Template-assisted	Yields well-defined nanorods with uniform dimensions and high reproducibility	Offers limited flexibility in adjusting the aspect ratio
	Allows precise control over the length and diameter of nanorods	Requires templates, which can be expensive and challenging to remove
	Potential for the synthesis of complex multi-segment nanorods with distinct properties	Template removal can be a cumbersome process
Photochemical methods	Offers a rapid and single-step synthesis process, minimizing time and effort	Provides limited control over aspect ratio, often resulting in lower aspect ratios and more spherical nanoparticles
	Minimizes the use of toxic chemicals, enhancing safety and reducing environmental impact	Scalability for large-scale production may be limited
	Suitable for *in situ* and on-site synthesis, making it versatile for various applications	May result in a wider range of shapes, including cubes and wires
Electrochemical methods	Simplicity and ease of use make it accessible for researchers with basic equipment	Generally provides limited control over aspect ratio and shape, often yielding more spherical nanoparticles
	Minimizes the use of hazardous reagents, enhancing safety	Scalability for large-scale production is limited
	Feasible for small-scale synthesis, suitable for laboratory settings	Requires specific electrodeposition setups
	Electrode degradation over time can affect reproducibility	Limited control over the length and diameter of nanorods

## 3 The physical meaning of optical tuning by aspect ratio and shape of (GNR)

Optical tuning by aspect ratio and shape of (GNR) refers to the ability to control and manipulate the optical features of these nanoscale structures by changing their aspect ratio (length-to-width ratio) and shape. (GNR) are elongated (GNPs) that exhibit unique optical characteristics because of the interaction among light and the collective oscillation of conduction electrons on their surfaces, known as (LSPR). Here are some key calculations related to the photophysical properties and quantification of gold nanorods:

Lifetime measurement:

The luminescence lifetime (*τ*) is the average time a fluorophore remains excited before returning to the ground state. Gold nanorods can be measured using time-resolved spectroscopy techniques like time-correlated single photon counting (TCSPC). The lifetime is then calculated by fitting the luminescence decay curve to an exponential function:*I*(*t*) = *I*_0_ exp(−*t*/*τ*)where *I*(*t*) is the intensity at time *t* and *I*_0_ is the initial intensity at *t* = 0.

Quantum yield calculation: the quantum yield (QY) measures the efficiency of the fluorescence process. For gold nanorods, it can be determined by:QY = (photons emitted)/(photons absorbed)

The number of photons emitted is obtained by integrating the emission spectrum. Photons absorbed is calculated using extinction coefficients and excitation intensity.

Plasmon decay rate:

The plasmon decay rate determines the lifetime of coherent localized surface plasmon oscillations in gold nanorods. It can be theoretically derived from plasmon bandwith in extinction spectrum using:*Γ* = hΔ*ω*where h is the reduced Planck's constant and Δ*ω* is the full width at half maximum of the plasmon peak.

These provide quantification of photophysical properties like lifetime and quantum efficiency as well as plasmon characteristics that influence nanorod optical sensing behavior. The LSPR of (GNR) strongly depends on their aspect ratio and shape, which may be precisely tailored during the synthesis process. By changing the aspect ratio, one can shift the resonance wavelength of the LSPR, thereby tuning the nanorods' optical properties. When the aspect ratio increases, the LSPR peak wavelength shifts to longer wavelengths, resulting in a redshift of the absorption and scattering spectra. Conversely, decreasing the aspect ratio results in a blueshift in the LSPR peak.

Additionally, the shape of (GNR) may be modified from a rod-like structure to more complex geometries, like triangular or hexagonal prisms. These variations in shape further enable precise control over the LSPR characteristics. The ability to tune the optical features of (GNR) by adjusting their aspect ratio and shape has noticeable implications for various uses. For example:

(1) *Sensing and detection*: the spectral tunability allows for the development of nanorod-based sensors that can detect and quantify target molecules or analytes through changes in the LSPR peak wavelength.

(2) *Imaging*: by selecting NRs with related aspect ratios and shapes, researchers can design contrast agents for various imaging techniques, including optical microscopy and biomedical imaging, to enhance resolution and specificity.

(3) *Photothermal therapy*: (GNR) can absorb light energy in the near-infrared region, where biological tissues are related and transparent. By tuning the LSPR to this region, NRs may be used for localized photothermal therapy, where light is heating transfer, select destroying cancer cells or pathogens.

(4) *Optoelectronics*: the ability to precisely control the optical features of NRs opens up possibilities for developing novel devices, like plasmonic waveguides, photodetectors, and solar cells, where the LSPR characteristics may be tailored for optimal performance.

In optoelectronics, the precise control of the optical features of gold nanorods (NRs) presents exciting opportunities for developing innovative devices. The unique plasmonic properties of NRs allow for the design of advanced components such as plasmonic waveguides, photodetectors, and solar cells. By tailoring the localized surface plasmon resonance (LSPR) characteristics of NRs, these devices can be optimized for superior performance. Plasmonic waveguides based on NRs enable the manipulation and guiding of light at the nanoscale, offering enhanced light confinement and propagation. This technology holds promise for applications in optical communication systems, sensing platforms, and integrated nanophotonic circuits.

Photodetectors incorporating NRs exhibit high sensitivity and tunable spectral response due to the strong plasmonic field enhancement. This enables efficient light detection across a broad range of wavelengths, making them valuable for imaging, optical sensing, and data communication. NRs in solar cells enhance light absorption and improve charge separation and transfer processes. The LSPR characteristics of the NRs can be precisely engineered to match the solar spectrum, resulting in increased energy conversion efficiency. The ability to precisely tune the optical properties of gold nanorods offers many opportunities for new technologies. Here are some specific applications you mentioned: plasmonic waveguides – by coating gold nanorods onto a substrate, researchers have created plasmonic waveguides that can guide and concentrate light at the nanoscale. This could enable highly integrated photonic circuits for data transmission and sensing. Ultrasensitive photodetectors – gold nanorods can act as plasmonic antennas that enhance light absorption and conversion into electrical signals. This effect can boost the sensitivity of photodetectors for applications like medical imaging and environmental monitoring. Solar cells – coating nanorods onto silicon solar cells has been shown to trap incoming sunlight and increase light absorption, potentially improving solar energy conversion efficiency. Plasmonic nanosensors have the potential to detect chemical and biological toxins in water efficiently. Gold and silver nanoparticles are commonly used as they exhibit localized surface plasmon resonance and have tunable optical properties.^130^ Their plasmon resonance depends on factors like shape, size and aggregation which can be altered upon interaction with analytes. This enables them to act as sensitive probes for detecting pollutants. Various techniques like colorimetry, UV-vis spectroscopy, surface enhanced Raman spectroscopy and electrochemical methods have been used with plasmonic nanosensors. They offer advantages like high selectivity, sensitivity, portability and ability for on-site detection. However, challenges remain in improving their reproducibility, stability and scalability for real world applications. Plasmonic nanosensors have been developed to detect chemical toxins like toxic metal ions, inorganic anions and organic pollutants. Gold and silver nanoparticles functionalized with different ligands show selectivity towards specific analytes. Similar nanosensors have also been used to detect pathogens like bacteria and biological toxins secreted by them. They offer rapid and sensitive detection as compared to conventional methods.

In summary, the tunable localized surface plasmon resonance of gold nanorods provides a flexible platform for enhancing light-matter interactions at the nanoscale. This opens up new possibilities for nanophotonics and nanoscale optoelectronics that offer performance gains in communication, imaging, and energy harvesting technologies. The precise control over nanorod synthesis enables optimization for specific applications, driving continued progress in this area.

Generally, optical tuning by aspect ratio and shape of (GNR) refers to manipulating their visual features by adjusting their length-to-width balance and overall condition. This tunability enables various sensing, imaging, therapy, and optoelectronics areas where precise control over the nanorods' optical response is crucial.

The (LSPR) band of (GNR) may be divided into two prominent bands: the longitudinal and transverse bands.

The longitudinal band is associated with the strong absorption and scattering of light in the (NIR) region. It arises from the collective oscillation of conduction electrons along the long axis of the nanorod. Because of this longitudinal oscillation, the LSPR peak of the nanorod is red-shifted significantly compared to that of spherical (GNPs). As the aspect ratio (length-to-width ratio) of the NRs increases, the longitudinal band experiences a pronounced redshift, resulting in a shift of the LSPR peak wavelength from the visible range to the NIR region. This shift is responsible for the color changes observed from blue to red as the aspect ratio of the NRs increases.

On the other hand, the transverse band is weaker in the visible range and has a wavelength close to that of (G) nanospheres. It arises from the collective oscillation of conduction electrons perpendicular to the long axis of the nanorod. The transverse band typically exhibits a weaker and less pronounced redshift than the longitudinal band.

By controlling the aspect ratio during the synthesis of (GNR), researchers can precisely tune the LSPR features and select to shift the absorption and scattering peaks to various regions of the electromagnetic spectrum. This tunability in the LSPR characteristics is valuable for multiple uses, like biomedical imaging, sensing, and photothermal therapy, where related wavelengths in the NIR region are advantageous because of improved tissue penetration and reduced background interference. The transverse band corresponds to the collective oscillation of conduction electrons perpendicular to the long axis of the nanorod. Unlike the longitudinal band, which experiences a noticeable redshift with an increasing aspect ratio, the transverse band remains relatively unchanged regarding wavelength position.

The insensitivity of the transverse band to size changes is primarily because the electron oscillations responsible for this band are mainly influenced by the width of the nanorod rather than its length. As a result, altering the aspect ratio by changing the nanorod's length while keeping the width constant does not substantially affect the transverse band's position.

Therefore, the ability to tune the optical features of (GNR) predominantly relies on controlling the longitudinal band through modifications in aspect ratio, while the transverse band remains relat unaffected. This various response allows for precise manipulation of the LSPR characteristics and the corresponding color changes observed in the longitudinal band.

These are some of the mathematical theories and concepts commonly used in the study of (GNR) like.

### 3.1 Mie theory

Mie theory is a mathematical theory that describes the scattering of electromagnetic waves by spherical particles. While (GNRs) are not spherical, Mie theory may be extended to approximate their optical features by treating them as prolate spheroids. The Mie theory describes the scattering of electromagnetic waves by spherical particles. The equation for the scattering efficiency (*C*_sca_) of a spherical particle may be expressed as:
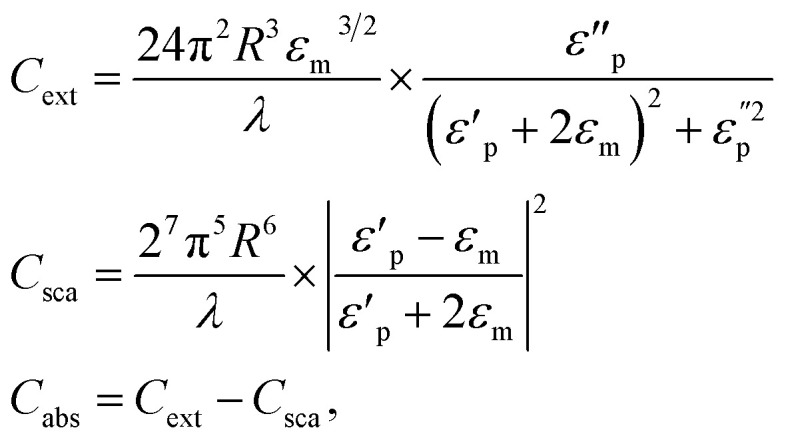
where *R* is the radius of the particle and *λ* is the incident wavelength.

Certainly, the Mie theory provides a comprehensive explanation of the interaction between light and nanoparticles, such as gold nanoparticles (AuNPs). In this context, the absorption and scattering probabilities are represented by the absorption cross section (*C*_abs_) and the scattering cross section (*C*_sca_), respectively, while the extinction cross section (*C*_ext_) is the sum of both (*C*_ext_ = *C*_sca_ + *C*_abs_).

These cross sections are calculated based on the principles of Mie theory, which involves solving complex mathematical equations to describe how light interacts with nanoparticles. Here's a simplified overview of how these cross sections is calculated. *Absorption cross section* (*C*_abs_): *C*_abs_ represents the probability that the AuNPs will absorb incident photons. To calculate *C*_abs_, Mie theory takes into account the size and shape of the AuNPs, the wavelength of incident light, and the refractive indices of both the AuNPs and the surrounding medium. The complex mathematical equations of Mie theory are solved to determine the absorption efficiency, and this efficiency is then used to calculate *C*_abs_ in square meters (m^2^). *Scattering cross section* (*C*_sca_): *C*_sca_ represents the probability that incident photons are scattered by the AuNPs. Like *C*_abs_, *C*_sca_ is calculated by Mie theory, considering the factors mentioned earlier. The scattering efficiency is determined through Mie theory, and this efficiency is used to calculate *C*_sca_ in square meters (m^2^). *Extinction cross section* (*C*_ext_): *C*_ext_ represents the overall interaction of AuNPs with incident light, encompassing both absorption and scattering. *C*_ext_ is simply the sum of *C*_sca_ and *C*_abs_ (*C*_ext_ = *C*_sca_ + *C*_abs_). By calculating these cross-sections using Mie theory, researchers can gain insights into how AuNPs interact with light at various wavelengths and under different conditions. This knowledge is essential for designing and optimizing applications involving AuNPs, such as in nanotechnology, sensing, and biomedical research, where controlling light interaction is crucial.

The Mie theory provides a mathematical framework for spherical particles to calculate various scattering features, like scattering cross-section, extinction cross-section, and scattering phase function. Please note that the Mie theory specifically applies to spherical particles, and for non-spherical particles like (GNR), modifications and extensions to the theory, like the Extended Mie Theory (EMT), are required to describe their optical properties accurately. One such extension is the T-matrix method, which can explain the scattering features of non-spherical particles, including (GNR). The T-matrix method for (GNR) involves solving equations to determine the T-matrix elements. The T-matrix relates the incident and scattered electromagnetic fields and may be used to calculate various scattering properties. The equations involved in the T-matrix method for (GNR) are complex and depend on the nanorods' related geometry and optical features. These equations consider factors like the nanorods' aspect ratio, the surrounding medium's dielectric characteristics, and the incident light's wavelength. It is recommended to refer to research papers or publications dedicated explicitly to the T-matrix method for (GNR) for a detailed understanding of the mathematical equations and modeling techniques involved. Please note that various numerical methods, like the Finite Element Method (FEM), Discrete Dipole Approximation (DDA), or other computational approaches, are often employed to solve the equations and calculate the scattering features of (GNR) accurately.

### 3.2 Maxwell's equations

Maxwell's equations are a set of fundamental equations in electromagnetism that describe the behavior of electric and magnetic fields. These equations are used to understand and model the interaction of light with (GNR).

Maxwell's equations are fundamental in classical electromagnetism and apply to all electromagnetic phenomena, including the interaction of light with (GNR). The equations themselves do not change specifically for (GNR) but provide a framework to describe the behavior of electric and magnetic fields in their presence. The interaction of (GNR) with electromagnetic fields may be characterized by incorporating the material features of (G) into Maxwell's equations. (GNR) exhibit plasmonic behavior because of the collective oscillation of conduction electrons on their surfaces, known as (LSPR). The LSPR phenomenon may be understood by considering the following modified Maxwell's equations:

1 Gauss's law for electric fields: ∇ × *E* = *ρ*/*ε*_0_

In the existence of (GNR), the charge density term (*ρ*) may include the impact of free charges induced on the nanorod surfaces or charge distributions associated with the plasmonic oscillations.

2 Gauss's law for magnetic fields: ∇ × *B* = 0

The magnetic field divergence remains zero, as no magnetic monopoles exist in the standard Maxwell's equations.

3 Faraday's law of electromagnetic induction: ∇ × *E* = −∂*B*/∂*t*

This equation describes electromagnetic induction, where changes in the magnetic field induce electric fields. The LSPR phenomenon in (GNR) can modify the induced electric fields because of the interaction with light.

4 Ampere's law with Maxwell's addition: ∇ × *B* = *μ*_0_*J* + *μ*_0_*ε*_0_∂*E*/∂*t*

In the existence of (GNR), the current density term (*J*) may account for the displacement current associated with the plasmonic oscillations or other electronic processes occurring within the nanorods.

### 3.3 Electromagnetic wave propagation

The behavior of light as it interacts with (GNR) may be described by the principles of electromagnetic wave propagation, including concepts like reflection, refraction, and diffraction.

The wave equation may describe the propagation of electromagnetic waves in (GNR), which is a solution of Maxwell's equations. The wave equation governs the behavior of electromagnetic fields as they propagate through space. Although the wave equation itself does not change specifically for (GNR), the material features of the NRs can influence the propagation characteristics of electromagnetic waves.

The wave equation for electromagnetic waves may be expressed as follows:∇^2^*E* − (1/*c*^2^)(∂^2^*E*/∂*t*^2^) = *μ*_0_∂^2^*P*/∂*t*^2^

In this equation:

• *E* represents the electric field vector.

• ∇^2^ is the Laplacian operator (second derivative concerning spatial coordinates).

• *c* is the speed of light in a vacuum.

• *t* represents time.

• *μ*_0_ is the vacuum permeability.

• *P* represents the polarization vector associated with the material response of the (GNR).

The existence of (GNR) affects wave propagation through their interaction with the incident electromagnetic field. The polarization vector (*P*) in the wave equation captures the response of the (GNR) to the applied electric field. The related form of the polarization vector depends on the model used to describe the optical features of the nanorods, like the Drude model or the Lorentz model.

To fully describe the electromagnetic wave propagation in the existence of (GNR), additional equations or models are required to characterize the material response of the NRs and their interaction with the incident electromagnetic field. These models consider factors like the plasmonic resonances, dispersion properties, and size-dependent impacts associated with the (GNR).

### 3.4 Plasmon resonance

Plasmon resonance refers to the collective oscillation of conduction electrons in a metallic nanoparticle in response to the interaction with incident light. Plasmon resonance plays a considerable role in determining the optical features of (GNR).

The LSPR may be modeled using the Drude–Lorentz model, which incorporates the response of the (GNR) to the incident electromagnetic field.

The equation that describes the plasmon resonance of (GNR) may be expressed as follows:*ω*_p_^2^ = *ω*_0_^2^ + (*Γ*/2)^2^

In this equation:

• *ω*_p_ represents the plasma frequency related to the collective oscillation of conduction electrons in the (GNR).

• *ω*_0_ represents the natural frequency of the plasmon resonance mode, which is determined by the nanorods' geometrical features (aspect ratio, size).

• *Γ* represents the damping constant or linewidth, which characterizes the decay rate of the plasmon resonance because of various loss mechanisms, including scattering, absorption, and radiative decay.

The plasma frequency *ω*_p_ is given by:*ω*_p_ = √(*N*_e_^2^/(*ε*_0_*m*_eff_))

In this equation:

• *N* represents the number density of conduction electrons in the (GNR).

• *e* represents the elementary charge.

• *ε*_0_ represents the vacuum permittivity (electric constant).

• *m*_eff_ represents the impact mass of the conduction electrons.

The plasmon resonance frequency *ω*_0_ depends on the geometrical features of the (GNR), like their aspect ratio (length-to-width ratio) and size. The damping constant *Γ* accounts for the broadening of the plasmon resonance because of various loss mechanisms.

The plasmon resonance of (GNR) is typically observed in the electromagnetic spectrum visible to the (NIR) region. By precisely controlling the aspect ratio and size of the nanorods, it is possible to tune the plasmon resonance wavelength, affecting the optical properties, like the nanorods' absorption, scattering, and extinction spectra.

### 3.5 Finite element method (FEM)

FEM is a numerical method used to solve various partial equations, including Maxwell's, for complex geometries. FEM may be employed to model and simulate the optical features of (GNR) by computer programs.

By incorporating Maxwell's equations, appropriate material properties, and boundary conditions, FEM programs can accurately predict the optical response of (GNR), including phenomena like absorption, scattering, and extinction. The numerical simulations enable researchers to investigate the impact of various parameters, like aspect ratio, size, and incident light properties, on the optical features of (GNR).

FEM-based programs for modeling (GNR) typically allow users to define the geometry of the nanorods, specify material properties, set up the simulation parameters, and analyze the results. These programs utilize advanced numerical techniques to solve the discretized equations and provide visual representations or quantitative data related to the optical features of the nanorods (Table 5).

**Table 5:** Summarizing the physical meaning, significance, and preferences of different theories and equations related to the optical tuning of gold nanorods (GNRs) by aspect ratio and shape

Theory/equation/concept	Physical meaning	Significance	Preferred?	Why?
Mie theory	Describes light scattering by spherical particles^131^	Fundamental theory for nanoparticle optics, but limited for nanorods	No	Only valid for spherical particles^132^
Gans theory	Extension of Mie theory for spheroidal particles^133^	Accounts for rod shape of GNRs	Yes	More accurate optical modeling of GNRs^134^
Maxwell's equations	Govern electromagnetic wave propagation and light-matter interactions^135^	Fundamental basis for describing GNR optics	Yes	Essential for modeling GNR interactions with light^136^
Plasmon resonance modeling (*e.g.* Drude model)	Describes resonant oscillations of conduction electrons^137^	Explains the origin of localized surface plasmon resonance (LSPR) in GNRs	Yes	Crucial for understanding tuning of LSPR by aspect ratio^138^
Finite element method (FEM)	Numerical technique to solve Maxwell's equations^139^	Allows computational modeling and simulation of GNR optical properties	Yes	Enables optimization and design of GNRs for applications^140^
Localized surface plasmon resonance (LSPR)	Collective oscillation of conduction electrons in response to light^141^	Determines the optical properties of GNRs	Yes	Tuning LSPR is key for optical tuning of GNRs^121^

## 4 Enhancing two-photon excitation efficiency through plasmonic nanostructures: a focus on (GNR) and near-field confinement

Gold nanomaterials with various morphologies, including (G) nanospheres, nanowires, nanoshells, nanocubes, nanoflowers, and nanotriangles. Among these morphologies, (GNR) stand out because of their peculiar optical features resulting from the (LSPR) impact. By regulating the synthesis of (GNR) with various aspect ratios, the longitudinal LSPR peaks may be tuned from visible light to the near-infrared region, enhancing the oscillator strength of the longitudinal LSPR. Plasmonic nanostructures, like (GNR), have shown great potential for strengthening two-photon excitation processes through near-field confinement. This section provides an overview of the current research on utilizing (GNR) to improve two-photon excitation efficiency. The unique features of (GNRs), including their tunable surface plasmon resonances and high local electromagnetic fields, make them ideal candidates for enhancing the excitation rate of nearby molecules or materials. This section discusses the mechanisms behind plasmon resonance-enhanced two-photon excitation, focusing on the interaction among conductive electrons in the (GNR) and electromagnetic fields. Enhancing two-photon excitation processes through plasmonic nanostructures, like (GNR), has opened up new possibilities in nanophotonics.^142^ This enhancement may be attributed to the strong local electromagnetic field near the nanostructures, which increases the two-photon excitation rate. The plasmon resonance of the nanostructures plays a considerable role in this enhancement, as it determines the wavelength at which the local electromagnetic field is maximized.^143^ By precisely controlling the size, shape, and composition of the (GNR), researchers can tune the plasmon resonance to match the excitation wavelength, resulting in a noticeable enhancement of the two-photon excitation process.^144^

Two-photon excitation is a valuable technique that allows the simultaneous absorption of two photons, resulting in the excitation of a molecule or material.^145^ This process offers advantages like reduced photodamage, increased spatial resolution, and deeper tissue penetration compared to traditional one-photon excitation methods. Plasmonic nanostructures, including (GNR), have emerged as promising tools for enhancing the efficiency of two-photon excitation through near-field confinement. The (LSPR) impact exhibited by (GNR) enables the manipulation of their optical properties, making them suitable for enhancing the excitation rate of nearby emitters. This review focuses on using (GNR) and near-field confinement to improve two-photon excitation efficiency and explores their potential uses in various fields.

**Fig. 10:**
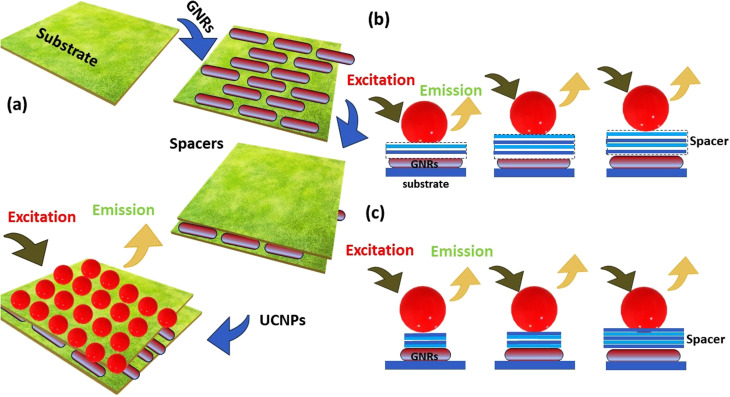
(a) A step-by-step guide for the fabrication of a novel UCNPs and (GNR) plasmon-enhanced fluorescence system, (b) examining UCNPs for distance-dependent plasmon-enhanced fluorescence: fine-tuning spacer thickness *via* layer-by-layer self-assembly and (c) achieving spectra-dependent plasmon-enhanced fluorescence: exploring various aspect ratios of (GNR).

Using (GNR) and near-field confinement offers exciting possibilities for various uses. In nanophotonics, plasmonic nanostructures can enhance the optical features of materials, enabling improved light-matter interactions and efficient energy transfer.^146^ In single-molecule spectroscopy, (GNR) acts as an antenna, concentrating the incident electromagnetic field and enhancing the excitation rate of weak emitters.^147^ This technique has enhanced noticeable fluorescence and can contribute to ultrasensitive detection and imaging advancements. In biomedical imaging, plasmonic nanostructures, including (GNR), have shown promise in improving contrast and imaging depth by enhancing two-photon-excited fluorescence. However, there are challenges associated with strengthening two-photon excitation through plasmonic nanostructures. Intense femtosecond pulses used for two-photon excitation can cause heating of the electron gas in the nanostructures, leading to the broadening of the plasmon resonance.^148^ This broadening can reduce the efficiency of the plasmon-enhanced two-photon excitation. Additionally, achieving precise positioning of a single emitter concerning the near field of the plasmonic structures may be challenging.^149^ Generally, plasmonic nanostructures, like (GNR), offer exciting opportunities for enhancing two-photon excitation processes and advancing fields like nanophotonics, single-molecule spectroscopy, and biomedical imaging. Continued research and development in this area hold promise for further improving the efficiency and applicability of plasmon resonance-enhanced two-photon excitation. A plasmon-enhanced fluorescence system was developed using upconversion nanoparticles (UCNPs) and (GNR) to generate ultrabright fluorescence bullets. The plan was designed to be easily fabricated without expensive equipment or complex procedures. In Fig. 10, the fabrication process involved using polyelectrolyte multilayers as spacers among the UCNPs and (GNR). These multilayers comprised posit charged polyallylamine hydrochloride (PAH) and negative charged sodium polystyrene sulfonate (PSS). The layer-by-layer self-assembly method was employed, which allowed for precise control over the thickness of the dielectric spacer at the nanometer scale. The distance among the UCNPs and (GNR), as plasmon nanostructures, was modulated by changing the thickness of the polyelectrolyte layers. This precise control over the spacer thickness enabled the investigation of plasmon-enhanced fluorescence. (GNR) with various aspect ratios used to achieve the spectra-dependence behavior of plasmon-enhanced fluorescence. These (GNR), named (GNR) −980, (GNR) −915, and (GNR) −735, had aspect ratios of 7.6, 6.3, and 4.5, respect.^150,151^ The plasmon-enhanced fluorescence system, comprising the (GNR)/polyelectrolytes/UCNPs trilayer structures, demonstrated the ability to generate ultrabright fluorescence bullets. The appropriate combination of UCNPs and (GNR) and the controlled spacer thickness enabled enhanced fluorescence. To explain the upconversion and downconversion properties of GNRs. Upconversion refers to the process by which lower-energy photons (typically in the near-infrared range) are absorbed by a material and converted into higher-energy photons, such as visible or ultraviolet light. GNRs can exhibit upconversion properties when combined with appropriate materials, such as rare-earth ions (*e.g.*, lanthanide-doped nanoparticles). This property allows GNRs to convert deep-tissue-penetrating near-infrared light into visible or UV light, which can have various applications in imaging and therapy. Downconversion, on the other hand, involves the conversion of higher-energy photons into lower-energy ones. While GNRs do not typically exhibit downconversion properties, they can be part of composite materials that incorporate other nanoparticles or molecules capable of downconversion. These composites can be designed to convert high-energy photons (*e.g.*, UV or blue light) into lower-energy photons (*e.g.*, visible or near-infrared light), which can be helpful for specific biomedical applications. The unique optical properties of GNRs and their hybrids, including upconversion and downconversion abilities, have significant implications for cancer treatment:

(1) GNRs are widely used in PTT, a minimally invasive cancer treatment method. When GNRs are exposed to near-infrared light (NIR), they efficiently absorb it due to their surface plasmon resonance (SPR) properties. This absorbed energy is converted into heat, leading to localized hyperthermia within cancer cells. This temperature rise can destroy cancer cells or sensitize them to other therapeutic agents.

(2) GNRs can also be employed in PDT. In this approach, GNRs are loaded with photosensitizing agents that generate reactive oxygen species (ROS) when exposed to specific wavelengths of light. ROS are highly toxic to cancer cells and can induce cell death. The upconversion properties of GNRs can be utilized to excite photosensitizers, allowing for deeper tissue penetration and targeted therapy.

(3) GNRs with upconversion properties are valuable in imaging applications. They can be used as contrast agents in fluorescence imaging or as carriers for fluorescent markers, allowing for precise cancer cell visualization. Downconversion materials within GNR composites can also enhance fluorescence-based diagnostics.

(4) GNRs can serve as drug carriers in cancer therapy. Their high surface area and tunable surface chemistry make them suitable for attaching therapeutic agents. Controlled release of drugs or other bioactive molecules can be achieved by triggering drug release through NIR irradiation.

In summary, the upconversion and downconversion properties of GNRs, either alone or in composites, make them versatile tools for cancer treatment. They enable targeted therapy, enhance imaging and diagnostics, and offer the potential for personalized and minimally invasive approaches to combat cancer. These unique optical properties continue to drive innovation in cancer research and treatment strategies.

Tumors often create a unique microenvironment characterized by acidic conditions (low pH). This acidity results from increased glycolysis, poor blood supply, and the accumulation of lactic acid. A stable PL emission in this acidic environment is essential for tumor imaging and diagnostics applications. PL-based imaging and diagnostic techniques, such as fluorescence imaging and biosensors, rely on the emission of fluorescent probes. Inconsistent PL emission under varying pH conditions can lead to inaccurate results, hampering the reliability of cancer diagnostics. In addition to diagnostics, stable PL emission is vital for targeted drug delivery and therapy monitoring. Nanoparticles or drug carriers with PL properties can be used to track drug release and monitor therapeutic effects within the tumor. Stable emission ensures accurate tracking and assessment. Many researchers are developing multifunctional nanoparticles that combine PL properties with therapeutic capabilities. These nanoparticles can deliver drugs, provide imaging contrast, and monitor treatment response simultaneously. Ensuring stable PL emission is critical for these multifaceted applications. Some innovative systems are designed to exploit the tumor's acidic pH. pH-responsive nanoparticles can release therapeutic agents or change their optical properties in response to the local pH environment. Stable PL emission is fundamental for the effectiveness of such systems. Experimental techniques used to study plasmon resonance-enhanced two-photon excitation include:

(1) *Two-photon excited luminescence (TPL) spectroscopy*: this technique measures the TPL intensity of a sample excited by a femtosecond laser, which may be used to study the enhancement of two-photon excitation by plasmonic nanostructures.

(2) *Scanning electron microscopy (SEM)*: SEM may be used to image plasmonic nanostructures and to study their morphology and size distribution.

(3) *Coherent control*: coherent control is an experimental technique that may be used to manipulate the phase and amplitude of the incident light to control the excitation of plasmonic nanostructures and to study the enhancement of two-photon excitation.

(4) *Quantum chemistry*: quantum chemistry can account for surface electron excitation, transport, transfer, and their influence on the optical features of molecules in plasmonic materials and structures.

(5) *Plasmon-enhanced TPL of single QDs by an individual (GNR)*: this technique involves (GNR) to enhance the TPL of single quantum dots, which may be used to study the enhancement of two-photon excitation.

(6) *Plasmonic enhancement of two-photon excited luminescence of (G) nanoclusters*: this technique involves the use of plasmonic nanostructures to enhance the two-photon excited luminescence of (G) nanoclusters, which may be used to study the enhancement of two-photon excitation.

The enhancement factor of plasmonic nanostructures may be measured in experiments using various techniques, including:

(1) *Field enhancement factor measurement*: this technique uses analytical solutions to measure the field enhancement factor at the surface of the plasmonic nanostructures.

(2) *Surface-enhanced Raman scattering (SERS)*: in this method, the plasmonic enhancement is reported by detecting the SERS of probe molecules inside a nanoantenna's gap area. The electromagnetic enhancement dominates the measured SERS enhancement factor (EF), which roughly corresponds to the fourth power of the local electric field enhancement.

(3) *Calculation of the plasmon resonant scattering peak location and enhancement factor*: this technique uses the local model to determine the peak positions and enhancement factor for gaps around 0.1 nm and 10 nm.

(4) *Electromagnetic (EM) enhancement features measurement*: this technique involves studying the EM enhancement features of plasmonic nanostructures by probing SERS spectra.

(5) *Confined electromagnetic field measurement*: this technique involves measuring the confined electromagnetic field in sub-wavelength dimensions produced by the light irradiation of plasmonic nanostructures.

(6) *Magnetic tuning of plasmonic excitation*: this technique involves preparing (GNR) with aspect ratios of 1.5–10 for which the surface plasmon absorption maxima are between 600 and 1300, and measuring the magnetic tuning of plasmonic excitation.

Measuring the enhancement factor of plasmonic nanostructures may be challenging because of the following reasons:

(1) Gap distances must be precisely controlled to the subnanometer length scale in three dimensions to investigate the plasmonic enhancement in a nanogap antenna using surface-enhanced Raman scattering (SERS). This is a challenge for nanofabrication and characterization techniques.

(2) Difficulty in direct measurements of near-field enhancement small scales, direct measures of near-field enhancement are still challenging., making it challenging to determine the enhancement factor of plasmonic nanostructures.

(3) Lack of a universal method for calculating enhancement factors: no universal method for calculating enhancement factors makes it difficult to compare results obtained using various techniques.

(4) Dependence on the features of the probe molecule: the enhancement factor of plasmonic nanostructures can depend on the parts of the probe molecule used in SERS experiments, making it challenging to compare results obtained using various probe molecules.

(5) Fabrication and characterization challenges: fabrication and characterization challenges can make it difficult to precisely control the size, shape, and morphology of plasmonic nanostructures, which can affect their enhancement factor.

Standard techniques used to fabricate plasmonic nanostructures include:

(1) *Electron beam lithography (EBL)*: EBL is a top-down technique that uses a focused beam of electrons to pattern a resist layer on a substrate, which is then used as a template for the deposition of metallic nanostructures.

(2) *Focused ion beam (FIB) lithography*: FIB lithography is a top-down technique that uses a focused beam of ions to pattern a resist layer on a substrate, which is then used as a template for the deposition of metallic nanostructures.

(3) *Dip-pen lithography (DPL)*: DPL is a bottom-up technique that uses an atomic force microscope (AFM) tip to deposit molecules onto a substrate to create patterns, which can then be used as a template for the deposition of metallic nanostructures.

(4) *Laser interference lithography (LIL)*: LIL is a top-down technique that uses a laser beam to create an interference pattern on a resist layer on a substrate, which is then used as a template for the deposition of metallic nanostructures.

(5) *Soft lithography*: soft lithography is a bottom-up technique that uses elastomeric stamps to transfer patterns onto a substrate, which can then be used as a template for the deposition of metallic nanostructures.

(6) *DNA-assisted lithography*: DNA-assisted lithography is a bottom-up technique that uses DNA molecules to create patterns on a substrate, which can then be used as a template for the deposition of metallic nanostructures.

These techniques can fabricate plasmonic nanostructures with various shapes and sizes, which may be used in multiple uses, including sensing, imaging, and optoelectronics. (GNR) and near-field confinement own emerged as powerful tools for enhancing the efficiency of two-photon excitation processes. The tunable features of (GNR) and compatibility with wet-chemical synthesis and surface functionalization provide opportunities for tailoring their plasmonic impact *s*. Despite challenges related to plasmon broadening and precise emitter positioning, advancements in this field offer exciting prospects for uses in nanophotonics, single-molecule spectroscopy, and biomedical imaging. Further research is needed to optimize plasmonic nanostructures, develop new strategies for emitter-nanorod coupling, and explore novel uses in other scientific and technological.

### 4.1 Significance of plasmonic nanostructures for improving two-photon excitation efficiency

Plasmonic nanostructures, including gold nanorods (GNRs), have gained significant attention in photonics and nonlinear optics due to their remarkable ability to enhance the efficiency of two-photon excitation (TPE). Here's a discussion of the significance of plasmonic nanostructures for improving TPE efficiency:

(1) Enhanced excitation rate:

Plasmonic nanostructures, such as GNRs, can significantly enhance the excitation rate of nearby molecules or materials through a phenomenon known as localized surface plasmon resonance (LSPR). When the LSPR of GNRs is tuned to match the excitation wavelength, a strong local electromagnetic field is enhanced near the nanostructures. This intense electromagnetic field increases the likelihood of simultaneous absorption of two photons, a key characteristic of TPE.

(2) Reduced photodamage:

TPE is advantageous over traditional one-photon excitation methods because it allows for excitation at longer wavelengths. Longer wavelengths penetrate tissues more effectively and cause less photodamage to biological samples. Plasmonic nanostructures like GNRs enable the efficient use of TPE for deep tissue imaging and biological applications by enhancing the excitation efficiency without increasing the incident laser power.

(3) Spatial resolution improvement:

Plasmonic nanostructures can confine the excitation field to extremely small volumes, resulting in improved spatial resolution in imaging and spectroscopy techniques. GNRs allow for precise targeting and selective excitation of molecules or structures within a sample by concentrating the excitation energy to specific regions. This is particularly valuable in techniques like super-resolution microscopy.

(4) Deep tissue imaging:

Plasmonic nanostructures enhance TPE efficiency in the near-infrared (NIR) region, where biological tissues are relatively transparent. This enables deep tissue imaging, making it possible to visualize structures and processes within living organisms with minimal interference from tissue scattering and absorption.

(5) Single-molecule sensing:

Plasmonic nanostructures act as antennas, concentrating incident electromagnetic fields at their surfaces. This effect is highly advantageous for single-molecule sensing and detection. GNRs can significantly amplify the fluorescence signals of individual molecules, enabling ultrasensitive detection and quantification of analytes. This has applications in fields such as biosensing and diagnostics.

(6) Biomedical applications:

Plasmonic nanostructures, including GNRs, have applications in various biomedical areas, such as cancer diagnostics and therapeutics. GNRs can be functionalized with targeting ligands and imaging agents, making them ideal for targeted drug delivery and imaging of cancer cells. Their ability to enhance TPE efficiency improves the sensitivity and specificity of these applications.

## 5 Synergistic (GNR)-therapy (SGNRT): targeting MDR tumors

Synergistic (GNR)-Therapy (SGNRT) is a promising approach in the field of cancer treatment, particularly for targeting multidrug-resistant (MDR) tumors. (GNR) are nanoscale structures made of (G) atoms arranged rod-like. They possess unique optical and physical features that make them attractive for various biomedical uses. In cancer therapy, SGNRT involves using (GNR) as a multifunctional platform for targeting and treating MDR tumors. The synergistic aspect refers to combining various therapeutic modalities to enhance treatment efficacy. Here's an overview of how SGNRT targets MDR tumors. (i) *Targeting*: (GNR) may be functionalized with related targeting ligands, like antibodies or peptides, that recognize and adhere to receptors overexpressed on the MDR tumor cells surface. This allows for the selective accumulation of (GNR) within the tumor site while minimizing off-target impacts. (ii) *(PTT)*: (GNRs) exhibit a phenomenon known as the (LSPR), where they absorb and convert (NIR) light into heat. By irradiating the tumor site with a NIR laser, the (GNR) absorbs the light energy, rapidly heats up, and induces hyperthermia select within the tumor cells. This localized hyperthermia can lead to tumor cell death or sensitization of MDR tumor cells to other treatment modalities. (iii) *Drug delivery*: (GNR) can serve as carriers for delivering chemotherapeutic drugs to the tumor site. The GNR surface may be modified to attach drug molecules or loaded with drug-loaded nanoparticles. Upon reaching the tumor, the drug may be released either by passing (because of the tumor microenvironment) or acting (by external triggers like light or heat). (iv) *Enhanced drug release and cellular uptake*: the localized hyperthermia generated by PTT can improve drug release from the (GNR) and enhance cellular uptake by MDR tumor cells. Elevated temperatures in the tumor microenvironment can disrupt drug-loaded nanoparticles or increase the permeability of cell membranes, thereby facilitating drug delivery and overcoming drug resistance mechanisms. (v) *Combined therapy*: SGNT combines the photothermal impact of (GNR) with chemotherapy or other therapeutic modalities to achieve a synergistic impact. The hyperthermia induced by PTT sensitizes MDR tumor cells to chemotherapy, radiation therapy, or immunotherapy. Combining these treatment modalities can enhance tumor cell killing, overcome drug resistance, and potentially reduce the required drug dosage.

### 5.1 Emerging strategies to overcome multidrug resistance (MDR) with (GNR)-based therapies

Moreover, SGNRT represents a promising strategy for targeting MDR tumors by combining the unique features of (GNR) with complementary therapeutic approaches. However, it's paramount to note that this field of research is still evolving, and further studies are required to optimize the SGNRT approach for clinical translation. This section highlights the use of (GNR) as a delivery system, a common theme among the following studies, either alone or in combination with other agents. Additionally, incorporating targeted cancer therapy, like related ligands or pH-responsive coatings, enhances treatment efficacy and minimizes off-target impacts. These studies highlight the importance of personalized and multifunctional approaches in cancer treatment. By combining various therapeutic modalities, like chemotherapy, phototherapy, and targeted therapy, the researchers aim to improve treatment outcomes and overcome multidrug resistance. The *in vivo* evaluations demonstrate enhanced tumor inhibition, improved anti-tumor ability, increased drug intracellular accumulation, and inhibited tumor metastasis. These studies provide valuable insights into developing innovative and impactive strategies for targeted cancer therapy and offer promising prospects for improving cancer treatment outcomes.

An example used system exhibited synergistic impact is carried by.^152^ The purpose of the study was to explore the synergistic impact of ROS. (PTX) and curcumin (CUR) on multidrug-resistant MCF-7/ADR cells utilising a system for the exact co-loading, co-delivery, and intracellular co-release of both drugs using (GNR). To create dual-drug conjugated biotin-PEG modified (GNR) (PTX/CUR@BPGNRs), the researchers attached PTX and CUR onto the same (GNR) in various ratios. This technique enabled the co-loading, co-delivery, and intracellular co-release of the two medicines in a predefined ratio. The researchers assessed the synergistic impact of PTX and CUR at various ratios. Compared to other ratios, the PTX/CUR@BPGNRs with a mass ratio of PTX and CUR at 1 : 1 displayed the strongest synergistic impact on MCF-7/ADR cells. The strongest synergism was seen in the free PTX and CUR mixture with a mass ratio 1 : 0.75. According to cytotoxicity experiments, PTX/CUR@BPGNRs killed MCF-7/ADR cells more impact *l* than the free drug combination. This suggests that the co-delivery of PTX and CUR to (GNR) increased their efficacy in combating multidrug resistance. When MCF-7/ADR cells were subjected to near-infrared irradiation, PTX/CUR@BPGNRs dramatically induced apoptosis. This method may be used for controlled drug release and focused therapy. The researchers found that PTX/CUR@BPGNRs significantly inhibited the expression of *P*-glycoprotein (*P*-gp) in MCF-7/ADR cells. *P*-gp is known to play a role in multidrug resistance, and its inhibition could enhance the impact *l* of chemotherapy. Generally, the study demonstrates the successful development of a system for precise co-loading, co-delivery, and intracellular co-release of PTX and CUR using (GNR). The system exhibited synergistic impacts, enhanced cytotoxicity, induction of apoptosis, and inhibition of *P*-gp expression in MCF-7/ADR cells. Methoxy-polyethylene-glycol-coated-(G)-NRs (MPEG-AuNR) and VER-155008-micelles were used in the study by (157) to increase the heat sensitivity of tumor cells and explore the therapeutic outcome of mild-temperature (PTT). A therapeutic system was created by the researchers by fusing MPEG-AuNR with VER-155008-micelles. They assessed VER-155008's impact on tumor cells' heat sensitivity and looked at the therapeutic impact of mild-temperature PTT paired with VER-155008-micelles. It was discovered that VER-155008-micelles reduced the production of heat shock proteins and decreased the heat resistance of tumor cells. HCT116 cells treated with VER-155008-micelles at 45 °C had similar *in vitro* survival rates to those treated with high-temperature hyperthermia (55 °C). By using photoacoustic imaging and fluorescence imaging, it was demonstrated that MPEG-AuNR and VER-155008-micelles both accumulated at the tumor location. In contrast to individual mild-temperature PTT (around 500 nm^3^) or control therapy with normal saline (greater than 2000 nm^3^), the combination of mild-temperature PTT (45 °C) with VER-155008-micelles caused a considerable decrease in tumor size (lower than 100 mm^3^ or gone). The findings showed that VER-155008-micelles reduced tumor cells' heat resistance and improved the therapeutic impact of mild-temperature photothermal therapy. According to the study, adding MPEG-AuNR to VER-155008-micelles boosted the therapeutic impact of mild-temperature photothermal therapy by increasing the heat sensitivity of tumor cells. The VER-155008-micelles down-regulated heat shock proteins, increased the heat-sensitivity of tumor cells, and resulted in a noticeable reduction in tumor size when combined with mild-temperature PTT. The study by,^153^ aims to develop (GNR) conjugated with a pH-sensitive zwitterionic polypeptide for combined chemo-photothermal therapy in treating cervical cancer. The researchers synthesized (GNR) with pH-sensitive zwitterionic polypeptide *via* a (G)-thiol interaction. The conjugates incorporated the anticancer drug doxorubicin (DOX) using an acid-labile hydrazone bond for pH-sensitive drug release in the tumor environment. The (GNR) conjugates were designed to undergo a surface charge conversion from negative to positive in the tumor extracellular environment, facilitating cellular uptake through electrostatic interaction. Once internalized by cancer cells, the hydrazone bond in the conjugates would be cleaved under the acidic intracellular environment, leading to the release of DOX. The (GNR) conjugates acted as impactive (NIR) photothermal materials, absorbing NIR photo energy and converting it into heat under irradiation. This photothermal impact efficiently killed tumor cells. The (GNR) conjugates exhibited excellent biocompatibility with normal cells, enhanced cancer cell uptake, and remarkable cancer cell-killing impacts in cell assays. In mice bearing HeLa tumors, the (GNR) conjugates demonstrated enhanced tumor inhibition efficacy through the combination of chemo-photothermal therapy. Generally, the study successfully developed (GNR) conjugated with a pH-sensitive zwitterionic polypeptide for combined chemo-photothermal therapy in cervical cancer treatment. The conjugates showed pH-triggered drug release, efficient cellular uptake, and impactive tumor cell killing through drug delivery and photothermal therapy. The results demonstrated the potential of these (GNR) conjugates for improved therapeutic outcomes in cervical cancer treatment. The study,^154^ is aimed to develop a multi-therapeutic nanotool by combining the photothermal features of (GNR) (AuNRs) with the photodynamic activity of the photosensitizer verteporfin for the treatment of colon cancer. The researchers coated AuNRs with natural materials, lipoic acid and (GG), to produce AuNRs_LA, GG. Verteporfin was loaded onto the coated AuNRs, resulting in stable colloidal dispersions called AuNRs_LA,GG/Vert. The stability, size, and morphology of the AuNRs_LA,GG/Vert were characterized. The hyperthermia impact after (NIR) excitation (810 nm) was evaluated to assess the increase in drug release profile in tumor-like media. Cytotoxicity studies were performed on the human colon cancer cell line HCT116. *In vivo* studies were conducted on HCT116 murine xenograft models to evaluate the ability of AuNRs_LA,GG to inhibit tumor growth through NIR laser-triggered hyperthermia. The combined photothermal (PTT) and photodynamic (PDT) impacts were demonstrated by administering AuNRs_LA, GG/Vert. The AuNRs_LA, GG/Vert colloidal dispersions showed stability, appropriate size, and morphology. NIR excitation induced hyperthermia, which increased the drug release profile in tumor-like media. The cytotoxicity studies demonstrated the efficacy of the combined PTT and PDT approach using AuNRs_LA, GG/Vert on HCT116 cells. *In vivo* studies showed that AuNRs_LA, GG impact arrested tumor growth through NIR laser-triggered hyperthermia. Furthermore, the administration of AuNRs_LA, GG/Vert resulted in complete xenograft depletion through the combined PTT and PDT impact *s*. Finally, the study successfully developed a nanotool using verteporfin-loaded (GNR) for combined photothermal/photodynamic therapy of colon cancer. The results demonstrated the stability of the nanotool, the impact *l* of hyperthermia-induced drug release, and the synergistic PTT and PDT impact in inhibiting tumor growth and achieving complete xenograft depletion. These findings highlight the potential of this multi-therapeutic approach for colon cancer therapy.

The study,^155^ aimed to develop a nanocomposite to combat multidrug-resistant (MDR) colorectal cancer (CRC) by combining photothermal therapy (PPT) with chemotherapy and overcoming drug resistance. The researchers prepared a nanocomposite made of a core (GNR) core and a triple-layer coating made of (GNRs/mSiO_2_/PHIS/TPGS/DOX). Doxorubicin was produced and placed onto mesoporous silica-coated (GNR) (GNRs/mSiO_2_) (DOX). To increase intracellular drug accumulation by impact endo/lysosome escape, pH-responsive poly-histidine (PHIS) was coupled to the (GNR)/mSiO_2_ to overcome DOX resistance. d-Tocopherol polyethylene glycol 1000 succinate (TPGS) was built on the particle surface to improve medication intracellular retention by inhibiting *P*-glycoprotein. The (NIR) photothermal conversion was an extreme impact on lives. The drug release was pH and NIR-triggered, DOX intracellular accumulation was raised, and MDR SW620/Ad300 cells were more sensitive to the nanocomposite's cytotoxicity. Importantly, in SW620/Ad300 tumor-bearing mice, the nanocomposite demonstrated the most muscular anticancer activity without considerable systemic toxicity compared to other control groups with either chemotherapy or photothermal therapy alone. The nanocomposite with a triple-layer covering was successfully developed, demonstrating its potential to fight MDR colorectal cancer. The nanocomposite impasse combined photothermal treatment, chemotherapy, and strategies to overcome drug resistance. The results highlight the promising role of nanotherapeutic systems in treating MDR colorectal cancer. The study successfully designed a multi-functionalized nanocomposite with a (GNR) core and triple-layer coating, which exhibited enhanced photothermal conversion, pH and NIR-triggered drug release, increased drug accumulation, and potent antitumor efficacy against MDR colorectal cancer.

The study by,^156^ aimed to develop a multi-functional nano-system to overcome *P*-glycoprotein (*P*-gp)-mediated multidrug resistance (MDR) in cancer and improve the efficacy of chemotherapy. Doxorubicin (DOX) and BAY-1082439 (a PI3K-110/PI3K-110 inhibitor) were encapsulated inside biodegradable PLGA-SH nanoparticles (NPs), which were then grafted onto (GNR) (Au NRs) modified with FA-PEG-SH to create the tumor-targeting drug delivery nano-system known as PBDF. This nano-system was created to increase the impact of *P*-gp-mediated MDR reversal, target tumor cells, and suppress *P*-gp-overexpressed MDR tumors. *In vitro* tests showed that, as compared to free DOX and free BAY-1082439, PBDF NPs significantly improved the absorption of DOX, increased the activity of reversing MDR, suppressed cell proliferation, and produced S-phase arrest and apoptosis in KB-C2 cells. Additionally, *in vivo*, tests showed that PBDF NPs reduced the growth of KB-C2 tumours and enhanced DOX's anti-tumor impact. Significantly, the administration of PBDF NPs prevented KB-C2 cells from spreading to the liver and lungs of naked mice, and neither *in vitro* nor *in vivo* no overt damage was seen. A PI3K-110/PI3K-110/inhibitor and DOX were successfully coupled in the study's multifunctional nano-system (PBDF) to circumvent *P*-gp-mediated MDR and increase the anti-cancer impact. The PBDF nano-system showed enhanced drug uptake, improved reversal of MDR, inhibited tumor growth, and suppressed metastasis in a preclinical model. These findings suggest the potential of the nano-system in addressing MDR in cancer and improving chemotherapy outcomes. it is clear that the nano-system enhanced drug uptake, reversed MDR, inhibited tumor growth, and suppressed metastasis. This research provides valuable insights into the development of innovative strategies to combat multidrug resistance in cancer treatment. Table 6 comprehensively summarizes various therapeutic approaches and drug delivery systems employed in studies involving Gold Nanorods (GNRs) for cancer therapy. Additionally, it highlights the use of targeted cancer therapy and *in vivo* evaluations conducted in each study. This table is a valuable resource for understanding the diverse applications and outcomes of GNR-based cancer treatments.

**Table 6:** Provides an overview of the therapeutic approaches, drug delivery systems, targeted cancer therapy, and *in vivo* evaluation conducted in each above study using GNR

Study	Therapeutic approaches	Drug delivery systems	Targeted cancer therapy	*In vivo* evaluation
152	Chemo-photothermal therapy	(GNR) conjugated with biocompatible zwitterionic polypeptide	Cervical cancer	Enhanced tumor inhibition
157	Photothermal/photodynamic therapy	(GNR) loaded with verteporfin	Colon cancer	Improved anti-tumor ability
153	Combination therapy (chemo-photothermal)	Multi-functionalized nanocomposite constructed by (GNR) core with triple-layer coating	Multidrug-resistant colorectal cancer	Enhanced drug intracellular accumulation
154	Reversal of multidrug resistance (MDR)	Multi-functional nano-system combining PI3K-110α/β inhibitor and doxorubicin	*P*-glycoprotein-mediated MDR in cancer	Improved anti-cancer efficiency
155	Mild-temperature photothermal therapy	(GNR) together with HSP inhibitor-VER-155008 micelles	Colon cancer	Enhanced photothermal therapy
156	Reversal of multidrug resistance (MDR)	Tumor-targeting drug delivery nano-system PBDF	*P*-glycoprotein-mediated MDR in colon cancer	Inhibited tumor metastasis, no obvious toxicity

### 5.2 Harnessing the plasmonic properties of (GNR) for sonodynamic therapy

Focuses on developing smart nanostructured lipid carriers for the synergistic chemo/photothermal therapy of breast cancer. Okuyucu *et al.* (2023)^158^ utilized a Trojan-like approach by entrapping doxorubicin (a chemotherapy drug) and (GNPs) within the lipid carriers. This combination allows for enhanced therapeutic efficacy through the synergistic impact of chemo and photothermal therapies. The authors discuss using smart nanostructured lipid carriers (NLCs) to deliver doxorubicin and (GNPs) for breast cancer chemo/photothermal therapy. Developing a Trojan-like drug delivery system that can select target cancer cells and release the drug in response to an external stimulus, like photothermal therapy. The study showed that the smart NLCs could deliver doxorubicin and (GNPs) to breast cancer cells, resulting in synergistic chemo/photothermal therapy. The authors describe the fabrication process of the nanostructured lipid carriers and evaluate their physicochemical properties. AuNPs were prepared in three desired geometries (sphere, rod, and cube) shapes. They encapsulated doxorubicin (DOX) as an antitumor agent into biocompatible nanostructured lipid carriers (NLCs) with high encapsulation efficiency in chemo/photothermal synergetic therapy. The absorption spectra of Au nanoparticles were measured, and distinct peaks were observed for various particle shapes. Au nanospheres displayed a single peak at 529 nm, indicating their characteristic absorption wavelength. On the other hand, Au nanorods exhibited two peaks at 538 nm and 869 nm, indicating their unique absorption properties. Lastly, Au nanocubes displayed a single peak at 636 nm, indicating their related absorption wavelength. The obtained (AuNPs) with various geometries were examined in (PTT) application after being loaded into nanostructured lipid carriers (NLC) to enhance their stability and targeted delivery. The obtained data confirmed that the choice of AuNRs as the photothermal agent is particularly advantageous because of their unique plasmonic properties. AuNRs exhibit a strong absorption peak in the (NIR) region, typically around 808 nm. This makes them suitable for PTT, as NIR light can penetrate deep into tissues while minimizing damage to healthy cells. The absorbed NIR light by the AuNRs led to localized heating, which could be utilized to select and destroy cancer cells or tumors while minimizing damage to surrounding healthy tissue. They also investigate the *in vitro* cytotoxicity and cellular uptake of the carriers in breast cancer cells. In this study, the researchers evaluated the cytotoxicity of nanostructured lipid carriers (NLCs) loaded with various (GNPs) (AuNPs) geometries on MDA-MB-231 breast cancer cells. They also investigated the therapeutic impact of the anticancer drug doxorubicin (DOX) when loaded into NLCs. The results showed that the NLCs had negligible cytotoxicity on MDA-MB-231 cells, with only a slight decrease in cell viability of approximately 7%, even in laser irradiation. However, when DOX was loaded into the NLCs, a noticeable reduction in cell viability was observed. After 24 hours of treatment, DOX and DOX NLCs reduced cell viability by 40.5% and 41.7%, respectively. These differences became pronounced after 48 hours, with reductions in cell viability of 52.1% and 65.4% for DOX and DOX NLCs, respectively. This indicates that the sustained release of DOX from NLCs enhances its therapeutic impact compared to free DOX. Furthermore, the researchers investigated the cytotoxic impact of AuNPs with various geometries on MDA-MB-231 cells by using AuNPs-loaded NLCs without DOX. After 48 hours of treatment in the absence of laser irradiation, it was observed that AuNSs NLC, AuNCs NLC, and AuNRs NLCs reduced the cell viability percentage to approximately 99.6%, 101.3%, and 102.3%, respectively, compared to the control group with a cell viability percentage of approximately 128.0%. These findings indicate that regardless of the geometry, AuNPs exhibit cytotoxicity against breast cancer cells. These findings contribute to understanding the cytotoxicity and therapeutic impact of AuNPs with various geometries when loaded into NLCs. The results suggest combining AuNPs and NLCs can enhance the therapeutic efficacy of anticancer drugs like DOX and exhibit inherent cytotoxicity against cancer cells. Further studies are warranted to elucidate the underlying mechanisms and optimize the formulation of AuNPs-loaded NLCs for improved cancer therapy outcomes. Overall, this research contributes to the field of nanomedicine and highlights the potential of smart nanostructured lipid carriers for the targeted delivery of drugs and (GNPs) for breast cancer chemo/photothermal therapy. In summary, multiple plasmon bands in (GNR), and the single plasmon band in (G) nanocubes reflect the intrinsic relationship between particle geometry and the resulting plasmonic properties. Understanding these distinctions is considerable for harnessing the unique optical characteristics of various (G) nanoparticle shapes and advancing their uses in various fields.

Sonodynamic therapy (SDT) is a promising cancer treatment modality that involves the activation of sonosensitizers by ultrasound waves to generate reactive oxygen species (ROS) within cancer cells, leading to their destruction. Loke *et al.* (2023)^159^ focus on utilizing the unique features of (GNR) and their alginate coating to enhance the generation of reactive oxygen species (ROS) when stimulated by ultrasound waves. The alginate coating on the (GNR) serves multiple purposes. It enhances the stability and dispersibility of the nanorods, ensuring their efficient delivery to the target site.

### 5.3 Enhancing sonodynamic therapy with alginate-coated (GNR) nanosonosensitizers

Additionally, the alginate coating acts as a ROS generator upon ultrasound irradiation, further enhancing the therapeutic efficacy of SDT. The study showed that the alginate-coated (GNR) could generate ROS under ultrasound irradiation, impacting sonodynamic therapy for cancer. The study demonstrates the biocompatibility and therapeutic potential of alginate-coated (GNR) in cancer treatment. The researchers evaluated the cytotoxicity and ROS generation capacity of the NRs *in vitro* using cancer cells. The results showed that the NRs impact generated ROS upon ultrasound stimulation, leading to apparent cancer cell death. In addition, the study evaluated the *in vitro* sonodynamic therapy (SDT) impact of alginate-coated (GNR) (AuNRsALG) upon ultrasound irradiation, focusing on the viability of MDA-MB-231 cancer cells under various treatment conditions. The cells were seeded in 96-well plates at a density of 5000 cells per well and allowed to grow overnight. The MDA-MB-231 cells were co-incubated with varying concentrations of AuNRs^ALG^ (ranging from 0 to 300 μg mL^−1^ in DMEM) and irradiated with ultrasound at a frequency of 1.0 MHz and a duty cycle of 50% for 3 minutes. The ultrasound was applied to one well at a time. The cell viability was assessed using the MTT assay, which measures cell metabolic activity. To establish the cancer cell killing efficiency of AuNRs^ALG^ as sonodynamic agents, the cells were also subjected to a separate (PTT) using a 638 nm diode laser. The laser was operated at an intensity of 1.0 W cm^−2^ and irradiated for 3 minutes. The laser was positioned at a fixed distance of 30 cm from the cells to standardize the laser intensity. By comparing the cell viability results obtained from the SDT and PTT experiments, the researchers could evaluate the impact of AuNRs^ALG^ as sonodynamic agent in killing cancer cells. This experimental setup allowed them to assess the therapeutic potential of AuNRs^ALG^*in vitro* and investigate the combined impact of ultrasound and laser irradiation on cancer cell death.

Furthermore, the researchers investigated the therapeutic efficacy of the nanosonosensitizers *in vivo* using a tumor-bearing mouse model. The sonotoxicity of AuNRs^ALG^ was assessed at various power intensities of ultrasound (ranging from 0 to 1.5 W cm^−2^) using the MTT assay. At an intensity of 1.5 W cm^−2^, cytotoxicity was observed in untreated cells and cells treated with AuNRs^ALG^ for 3 minutes of ultrasound irradiation at 1.5 W cm^−2^ resulted in a noticeable reduction in cell viability, indicating that the observed cytotoxicity was primarily because of the heat generated during ultrasound irradiation. Lower ultrasound intensities of 0.5 W cm^−2^ and 1.0 W cm^−2^ did not cause sonotoxicity in untreated cells but reduced cell viability in MDA-MB-231 cells treated with 200 μg mL^−1^ AuNRs^ALG^. Based on these results, 1.0 W cm^−2^ of ultrasonic intensity was chosen for additional cell research. The dose-dependent sonotoxicity impact of AuNRs^ALG^ (ranging from 0 to 300 μg mL^−1^) at 1.0 W cm^−2^ was further evaluated using the MTT assay. Noticeable sonotoxicity was observed at 150 μg mL^−1^ of AuNRs^ALG^; the greatest cell death of 81.3% was achieved at 300 μg mL^−1^. The IC_50_ value of AuNRs^ALG^-mediated SDT was determined to be 136 μg mL^−1^. The cytotoxicity profiles of AuNRs^ALG^ activated by ultrasound were compared to those activated by visible light (638 nm laser irradiation). The *in vitro* cytotoxicity profiles of AuNRs^ALG^ + ultrasound treatment resembled those of AuNRs^ALG^ + laser treatment, with unnoticeable differences in cell death percentages.

Flow cytometry analysis was performed to study the cell death pattern induced by AuNRs^ALG^-mediated SDT. The proportion of viable cells decreased significantly in the AuNRs^ALG^ + ultrasound group compared to the untreated group, indicating a nearly 6-fold reduction in cell survivability. The percentages of early and late apoptotic cells increased, while the population of necrotic cells was negligible. Western blot analysis was conducted to understand the mechanism underlying AuNRs^ALG^-induced apoptosis. The cells treated with AuNRs^ALG^ + ultrasound showed a noticeable increase in the expression of phosphorylated H2A histone (γH2AX), a marker for double-strand breaks in DNA. The combined treatment also resulted in the downregulation of the anti-apoptotic protein Bcl-2, suggesting the involvement of the mitochondrial pathway in cell death. These results indicate that AuNRs^ALG^-mediated SDT induces cancer cell death primarily through apoptosis rather than necrosis. The results demonstrated that SDT with the alginate-coated (GNR) impact suppressed tumor growth and improved survival rates in the treated mice. The study highlights the potential of using ultrasound as an energy source for activating AuNRs^ALG^ and treating cancer and the possibility of ROS-generating alginate-coated (GNR) as biocompatible nanosonosensitizers for impact sonodynamic therapy of cancer, considering its better tissue penetration compared to visible light. Developing such nanosystems holds promise for improving cancer treatment outcomes by combining ultrasound stimulation and nanotechnology-based strategies.

### 5.4 Synergistic photothermal-chemotherapy using triple-combination nano-system for MDR tumors: an *in vivo* study

To evaluate the therapeutic efficacy of the multifunctional (GNR), both *in vitro* and *in vivo* experiments were conducted. In the *in vitro* experiments, tumor cells were treated with multifunctional (GNR) and exposed to (NIR) laser irradiation. The (GNR) was designed to induce a low-temperature photothermal impact upon NIR irradiation, disrupting tumor metabolism and inhibiting tumor growth. The (GNR) included siRNA components that facilitated the related knockdown of target genes, further suppressing tumor cell proliferation. In the *in vivo* experiments, the multifunctional (GNR) was administered to tumor-bearing mice, followed by NIR laser irradiation to induce the low-temperature photothermal impact. The researchers assessed the therapeutic outcomes by monitoring tumor growth, analyzing gene expression within the tumors, and evaluating the overall survival rates of the mice.

In the study conducted by Shin *et al.* (2023),^160^ the therapeutic efficacy of a triple-combination nano-system was assessed in an *in vivo* setting using mice bearing multidrug-resistant (MDR) tumors. The study aimed to develop a novel nano-system that combines (G) nanoclusters (AuNCs), quercetin (QU), and docetaxel (DTX) for synergistic photothermal-chemotherapy against multidrug-resistant (MDR) tumors. The researchers utilized RNA sequencing (RNA-Seq) to guide the selection of therapeutic agents and optimize their combination. AuNCs were functionalized with polyethylene glycol (PEG) to enhance their stability and biocompatibility. Quercetin, a natural compound with anti-cancer properties, was loaded onto the AuNCs through electrostatic interactions. Docetaxel, a commonly used chemotherapy drug, was encapsulated in polymeric micelles for improved solubility and targeted delivery. *In vitro* and *in vivo* experiments were conducted to evaluate the therapeutic efficacy of the triple-combination nano-system. MDR tumor cells were treated with the nano-system, and cell viability was assessed using various assays. The results demonstrated that the combined treatment of AuNCs, QU, and DTX exhibited superior cytotoxicity compared to individual or dual-component treatments, indicating a synergistic impact. To evaluate the photothermal effect of the AuNCs, (NIR) laser irradiation was applied to the MDR tumor cells treated with the nano-system. The AuNCs efficiently transfer NIR light into heat, leading to localized hyperthermia and enhanced therapeutic efficacy.

A (NIR) laser with a wavelength of 808 nm was used to examine the photothermal impact of the samples exposed to laser light. Each model (*n* = 3) was exposed separately to the NIR laser at a power of 1.2 W for 5 minutes to evaluate the photothermal impact as a function of time. They were causing a repeatable rise in temperature, reaching about 45 °C at the tumor location. When subjected to repeated laser irradiation, the quercetin/GNC combination treatment impact inhibited tumor growth compared to the group treated with (GNC) alone. Additionally, the combined therapy caused the tumor tissue to be more severely damaged. Notably, no noticeable pathological abnormalities or lesions were seen in the control group, demonstrating the efficacy of the therapeutic strategy. NIR thermographic pictures were collected at regular intervals to track the temperature changes. The material was allowed to cool after the initial NIR exposure before receiving additional laser radiation. For comparison, the (GNR) was employed as the control.

The following steps were used to create Chlorin e6 (Ce6) loaded in PEG-PLA micelles (Ce6-M) for *in vivo* fluorescent real-time tumor imaging. PEG-PLA polymer was dissolved in DCM, Ce6 was dissolved in EtOH, and the two solutions were combined in a 10 : 1 weight ratio. The thin films of the drug–polymer mixture are formed by evaporating the organic solvent under a stream of inert nitrogen gas and hydrating the thin film with distilled H_2_O (DW) by shaking. Using a UV-vis spectrophotometer at 663 nm and analysis in dimethylformamide, the amount of Ce6 in the PEG-PLA micelle was quantified (DMF). Ce6 was loaded with 92.8 percent impact into PEG-PLA micelles. Using free Ce6 in DPBS as well as Ce6-loaded micelles (Ce6-M), fluorescence real-time tumor imaging was carried out. With an equivalent dose of Ce6, both formulations were injected into the tail veins of mice carrying UV-2237 M tumors. Using a Fluorescence In Vivo Imaging System, the biodistribution of Ce6-M over time following injection was observed (FOBI System, Neo Science, Suwon, South Korea). Nude mice were euthanized 24 hours after injection, and the tumor and other organs were taken out for *ex vivo* examination of fluorescence levels, which showed the accumulation of Ce6-M. Compared to free Ce6, this investigation evaluated the dispersion and tumor-related accumulation of the Ce6-loaded micelles.

The study conducted by Fan *et al.* (2023)^161^ focused on the application of multifunctional (GNR) in low-temperature photothermal interactions for combined tumor starvation and RNA interference (RNAi) therapy. The researchers aimed to develop an innovative therapeutic approach that combines the advantages of photothermal therapy and RNAi to achieve enhanced tumor treatment outcomes. This study used two components—a polyethylene glycol (PEG) surface coating and small interfering RNA (siRNA) targeting relevant genes linked to tumor growth—to synthesize and functionalize the multifunctional (GNR). The siRNA component allowed for relevant gene silencing within the tumor cells, while the PEG coating increased the stability and biocompatibility of the (GNR). The following steps were involved in creating siRNA-GOx/GNR@HA NPs: I A modified seed-mediated growth technique was used to create the (GNR). After gently combining 0.25 mL of HAuCl4 (0.01 M) with 7.5 mL of CTAB solution (0.1 M), 0.6 mL of ice-cold NaBH4 was added. The mixture was maintained at 25 °C for two hours to create the seed solution. Separately, a test tube was filled with 4.75 mL of CTAB solution (0.1 M), 0.2 mL of HAuCl_4_ solution (0.01 M), and 0.03 mL of AgNO_3_ solution (0.01 M), which were all gently mixed. Then, 0.01 mL of the seed solution and 0.032 mL of (AA) (0.10 M) were added. The reaction was carried out at 37 °C in a dark environment for three hours. Centrifuging the resulting CTAB-stabilized (GNR) (GNR-CTAB), re-dispersion in distilled water, and UV-vis spectrophotometer confirmation were all done. (ii) The supernatant was discarded after centrifuging a 1 mL aliquot of the (GNR) solution. Preparing MPEI-SH functionalized (GNR) (GNR-PEI) involved combining (GNR) with MPEI-SH using a modified version of an earlier procedure. (iii) To create GOx-coated GNR-PEI (GOx/GNR NPs), 100 L of glucose oxidase (GOx) was combined with GNR-PEI ([Au] = 1 mg mL^−1^) and swirled for two hours. Centrifugation was used to separate the resultant residue, which was then subjected to three PBS washes. The supernatants were gathered using a BCA protein assay kit to measure the unconjugated or loosely bound GOx. The weight of dedicated GOx minus the weight of GOx in the supernatant, divided by the weight of all nanoparticles, was used to compute the loading content of GOx. (iv) GOx/GNR NPs were gently mixed with B7-H3si (10 OD mL^−1^ in DEPC–water) at a (G): a mass ratio of 2 : 1 to 100 : 1 for B7-H3si. Thirty minutes were spent incubating the mixture at ambient temperature to create siRNA and GOx coloaded (GNR) (siRNA-GOx/GNR NPs). (v) A gentle mixture of 1 mL of the obtained GNS/siRNA and 250 L of 20 mg mL^−1^ HA was stirred for 30 minutes to create siRNA-GOx/GNR NPs@HA. Centrifugation at 8000 rpm for 20 minutes purified and collected the resultant siRNA-GOx/GNR NPs@HA. They were then once again dissolved in distilled H_2_O before being used. Finally, (TEM) and (DLS) were used to investigate the particle size and morphology of the produced NPs. Using a PerkinElmer Optima-5300DV spectrometer, induct coupled plasma optical emission spectrometry (ICP-OES) was used to measure the amount of (G) in the NPs.

**Fig. 11:**
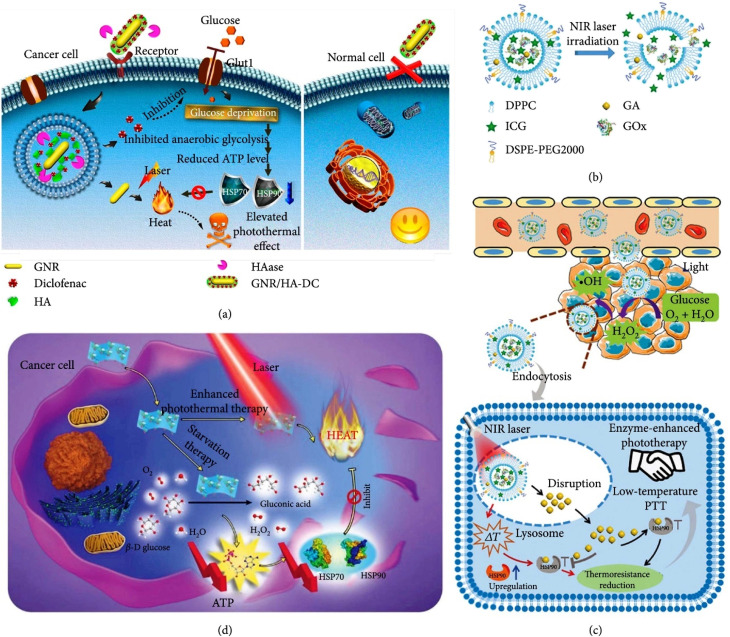
(a) Scheme showing the use of GNR/HA-DC to sensitize cancer cells to mild PTT by interfering with the anaerobic glycolysis metabolism. Reprinted with permission from Ref. 162. Copyright 2017, American Chemical Society. (b) Scheme illustrating the transformation of GOIGLs upon NIR light irradiation. (c) Scheme depicting the mechanism of EEPT and low-temperature PTT. (b and c) Reprinted with permission from Ref. 163. Copyright 2020, Wiley-VCH. (d) Scheme showing the utilization of M-NS for cancer starvation therapy and PTT. Reprinted with permission from Ref. 164. Copyright 2019, Wiley-VCH.

Eight tumor-bearing mice were created to assess the anticancer impact *in vivo*. Various intravenous injections were given to each group, including saline, siRNA, glucose oxidase (GOx), siRNA-GOx, siRNA/GNR NPs, siRNA/GNR@HA NPs, siRNA-GOx/GNR NPs, or siRNA-GOx/GNR@HA NPs. The GOx and B7-H3 siRNA doses were 2 mg kg^−1^ and 1 mg kg^−1^ concerning body weight. Thirty-six hours after injecting the various formulations, the mice in the subcutaneous A549 tumour model were subjected to an 808 nm laser for 5 minutes at a power density of 1.0 W cm^−2^. An infrared imaging spectrometer was used to capture (NIR) thermal images while measuring the tumor site's temperature (FOTRIC 220 s). In all groups, it was necessary to maintain a tumour site temperature below 45 °C. Every third day for 21 days, the tumour volume and mouse weight were measured. The B7-H3, HSP90, HSP70, and cleaved caspase 9 expression levels in tumor cells were determined through immunofluorescence and immunohistochemical staining. In addition, normal kidney, liver, lung, and other tissues were gathered, fixed, and stained with Hematoxylin–Eosin (H&E) under a microscope. Through these tests, the researchers could assess the impact of the various formulations for *in vivo* gene silencing and photothermal therapy, their impact on tumor growth and the expression of associated biomarkers in tumor cells. Fig. 11 illustrates various nanoplatforms and strategies used for cancer therapy, combining photothermal effects, enzyme-mediated therapy, and controlled drug release for enhanced treatment outcomes. Fig. 11a illustrates a nano platform consisting of gold nanorods (GNR) functionalized with hyaluronic acid (HA) and loaded with a drug (DC). The HA on the surface targets CD44-overexpressed tumor cells. GNR/HA-DC exhibits strong photothermal conversion ability, which means it can convert light (likely near-infrared) into heat. This property is advantageous for photothermal therapy (PTT) of CD44-overexpressed tumors. HAase in the tumor microenvironment (TME) helps release DC from the nanoplatform, which inhibits Glut1 in cancer cells. The nanoplatform can achieve cancer-specific mild-temperature PTT, ensuring minimal damage to healthy cells. Fig. 11b represents thermosensitive liposomes loaded with GA (likely gallic acid), GOx (glucose oxidase), and ICG (indocyanine green). Upon near-infrared (NIR) light irradiation, ICG generates heat within the liposomes. When the temperature crosses a certain threshold (above 42 °C), the liposomes undergo a phase transition, releasing the loaded GA, GOx, and ICG. Under visible light illumination, GOx catalyzes glucose oxidation, producing hydrogen peroxide (H_2_O_2_), which is then transformed into highly reactive hydroxyl radicals (˙OH). This contributes to enzyme-enhanced phototherapy (EEPT). After NIR light irradiation, ROS disrupts lysosomes, releasing GOx and GA into the cytoplasm. The combination of mild PTT and cancer starvation therapy (using GOx and GA) enhances the therapeutic effect. Fig. 11c emphasizes the concept of low-temperature photothermal therapy (PTT) using a nanoplatform. GOx consumes glucose, and GA downregulates heat shock proteins (HSPs) in cancer cells. After NIR light irradiation, ROS disrupts lysosomes, aiding the release of GOx and GA into the cytoplasm. The combined approach of mild PTT and cancer starvation therapy shows a robust anticancer therapeutic effect. Fig. 11d represents two-dimensional MnO2 nanosheets that exhibit GOx-like catalytic performance and NIR-absorbing ability. M-NSs consume intracellular glucose, leading to the downregulation of HSPs in cancer cells. Under NIR light irradiation, M-NSs generate heat, achieving low-temperature PTT. The nanosheets can be functionalized for further applications, such as imaging.

The study's outcomes showed the efficacy of multifunctional (GNR) in combination with tumor starvation and RNA interference (RNAi) therapy. The nanosystem was created using three various B7-H3 siRNA sequences. More than 94 percent of the cells were killed following laser irradiation, demonstrating the synergistic impacts of photothermal treatment (PTT), glucose deprivation-induced tumor starvation, and B7-H3 gene silencing. In contrast to non-targeted nanocarriers, the HA-coated nanocarriers significantly increased the cytotoxicity on A549 cells, highlighting the particular susceptibility of cancer cells to the nanosystem. siRNA-GOx/GNR@HA NPs showed the potential to lessen harm to CD44-negative normal cells by targeting CD44-overexpressing cancer cells, hence enhancing tumor formation and penetrating capacity. HSP90 and HSP70 expression was successfully downregulated by the loaded siRNA and GOx within siRNA-GOx/GNR@HA NPs. Because of the multifunctional low-temperature photothermal treatment impact, the administration of siRNA-GOx/GNR@HA NPs successfully reduced tumor growth and increased the survival duration of tumor-bearing mice. Therefore, this multifunctional low-temperature photothermal nanosystem showed more impact and relevant anticancer properties compared to high-temperature photothermal systems. It holds great potential for clinical application in tumor treatment, offering a promising approach to cancer therapy. The low-temperature photothermal impact generated by the (GNR) significantly inhibited tumor growth in both *in vitro* and *in vivo* models. Moreover, the targeted delivery of siRNA using the (GNR) led to related gene silencing, further enhancing the therapeutic impact.

The study conducted by Bemidinezhad *et al.* (2023)^165^ aimed to investigate the potential of (G)-containing liposomes and glucose-coated (GNPs) (GNPs) as radiation sensitizers for B16F0 melanoma cells. The researchers compared the impact of (G) ions-containing liposomes (referred to as (G)-lips) with glucose-coated (GNPs) (referred to as Glu-GNPs) in enhancing the radiosensitivity of melanoma cells. The study involved treating B16F0 cells with (G)-lips and Glu-GNPs at various concentrations and assessing their cytotoxicity when combined with 2 Gy irradiation. The researchers evaluated the radiosensitizing impact of the treatments and compared their efficacy. The results demonstrated that both (G)-lips and Glu-GNPs when combined with 2 Gy irradiation, impact enhanced the radiosensitivity of B16F0 cells at non-toxic concentrations of 10 and 15 μg mL^−1^, respect. This indicated that the (G)-based nanomaterials sensitized the melanoma cells to radiation-induced cell death.

Furthermore, the study revealed that (G)-lips exhibited a more remarkable ability to induce apoptosis and enhance radiation sensitivity than Glu-GNPs. This suggests that the (G) ions-containing liposomes had a more powerful impact in promoting cell death and increasing the radiosensitivity of B16F0 cells. Based on these findings, the researchers concluded that (G)-lips own promising potential as radiation sensitizers. They demonstrated radiosensitizing solid impacts and impact-induced apoptosis in B16F0 melanoma cells. The study suggests that further investigations, both *in vitro* and *in vivo*, are necessary to explore the application of (G) ions-containing liposomes in cancer treatment.

Basaran *et al.* (2023)^166^ highlight the potential of photothermal control using plasmonic (GNR) in engineered living materials, paving the way for advancements in targeted drug delivery, biosensing, virus capture, and bioremediation processes. The researchers investigated the potential of plasmonic stimulation using (GNR) to achieve precise control over ELMs. Plasmonic stimulation refers to the excitation of plasmons by external light sources, which are collective oscillations of electrons in a material. In this case, (GNRs) were used because of their unique optical properties, including a strong absorption peak in the (NIR) range. Commercial (GNR) (41 nm long, 10 nm diameter) stabilized with CTAB (cetyltrimethylammonium bromide) was used. Concentrations of CTAB and AuNRs were adjusted during centrifugation, which could increase the risks of accumulation. The agglomeration state of AuNRs in the GNC hydrogel was assessed using UV-vis absorbance spectroscopy and cryo-TEM (transmission electron microscopy). NRs with an (LSPR) at 808 nm were chosen to match the transmissive window of tissues and enable efficient photothermal heating. The photothermal conversion efficiency of the GNC hydrogels was quantified and found to be comparable to previous reports on AuNR dispersions in water. Bilayer hydrogels were prepared with the GNC hydrogel as the top layer and a pure hydrogel as the bottom layer. Illumination of the bilayer hydrogel with 808 nm light resulted in photothermal heating, where the (GNR) in the top layer absorbed light and transferred it into heat. Heating, cooling, and intermittent illumination studies were performed to evaluate the photothermal activity and stability of the bilayer hydrogel. Temperature measurements were performed using infrared thermometry and embedded thermocouples to compare surface and bulk temperatures. A linear correlation between surface and bulk temperatures was found, enabling the conversion of surface temperature measurements into bulk temperatures. The ability of the GNC hydrogel to stimulate mCherry-producing thermoresponsive bacteria was tested, demonstrating uniform fluorescence activation under certain laser power densities. mCherry is a red fluorescent protein commonly used as a genetic marker in molecular and cellular biology research. It belongs to the family of fluorescent proteins derived from Discosoma sp. coral, and its bright red fluorescence allows for easy visualization and tracking of cells and cellular processes. This study demonstrates the ability of (GNR)-loaded hydrogels to stimulate mCherry-producing thermoresponsive bacteria through photothermal impact *s*. By selecting and activating the bacteria using NIR light, researchers can control mCherry expression within the hydrogel. Such a system could own uses in biomedical research, tissue engineering, and drug delivery, where precise spatial and temporal control over cellular behavior and gene expression is desirable. To stimulate the mCherry-producing bacteria, the researchers applied (NIR) light, which matched the LSPR wavelength of the (GNR). The (GNR) absorbed and transferred the NIR light into heat, resulting in a localized temperature increase within the hydrogel. In this study, the researchers incorporated (GNR) into a hydrogel matrix and encapsulated mCherry-producing thermoresponsive bacteria within the hydrogel. The researchers incorporated (GNR) into a hydrogel matrix and encapsulated mCherry-producing thermoresponsive bacteria within the hydrogel. The hydrogel served as a scaffold to provide structural support and promote the interaction between the bacteria and the (GNR). They used fluorescence microscopy to detect the fluorescent signal of mCherry across the entire bacterial hydrogel. This imaging technique allowed them to spatially quantify the fluorescence intensities with a resolution as fine as 2 μm in the *XY* plane. The results indicate an increase in fluorescence intensity with each hour of photothermal stimulation. The researchers applied photothermal heating at a laser power density of 0.7 W cm^−2^, resulting in a uniform expression of mCherry. The corresponding temperature range on the silicon oil surface was 38–41.5 °C, with slightly higher temperatures observed in the bulk gel. This temperature range was ideal for achieving the greatest rate of gene expression possible while preserving bacterial viability. Additionally, the researchers found that adjusting the laser power density to 0.5–0.6 W cm^−2^ could achieve a surface temperature profile ranging from 35–37.5 °C. This temperature profile led to the greatest mCherry expression in the center of the bacterial hydrogel, gradually decreasing towards the periphery. These observations indicate the potential for locally confined activation of extracellular matrix-like (ELM) operations utilizing this system. By controlling the laser power and maintaining the same (GNR) concentration, The spatial profile in which the ELMs may be turned on can now be modified. In conclusion, the study demonstrated that photothermal stimulation using related laser power densities could activate the bacterial thermoresponsive switch, leading to the expression of mCherry. The researchers observed spatially controlled mCherry expression, allowing for precise manipulation of gene expression within the bacterial hydrogel system. These findings have implications for developing systems that can locally regulate cellular functions and offer potential uses in a range of domains in a wide array of disciplines, including tissue engineering and controlled drug delivery. Huang *et al.*^167^ developed a multifunctional hydrogel system that responded to (NIR) light for diabetic wound healing. The hydrogel incorporated peptide-functionalized (GNR) to release therapeutic agents sequentially upon exposure to NIR light. The goal was to address the challenges associated with diabetic wound healing by providing controlled drug release and promoting tissue regeneration. The researchers synthesized the peptide-functionalized (GNR) and embedded them within the hydrogel matrix. These NRs served as photothermal agents, capable of converting NIR light into localized heat, triggering the release of therapeutic agents from the hydrogel. The release of growth factors and antimicrobial peptides was achieved by controlling the duration and intensity of exposure to NIR light.

The study evaluated the performance of the hydrogel system *in vitro* and *in vivo* using a diabetic wound model. The results showed that the NIR light-responsive hydrogel impact released therapeutic agents in a controlled manner, promoting wound healing and inhibiting bacterial growth. The sequential release of growth factors and antimicrobial peptides provided a synergistic effect, enhancing the healing process and reducing the risk of infection. Overall, the study demonstrated the potential of the NIR light-responsive multifunctional hydrogel system for diabetic wound healing. By utilizing the photothermal features of (GNR) and the controlled drug release capability of the hydrogel, this approach offers a promising strategy to address the challenges associated with diabetic wounds, providing an innovative and impactful therapeutic option. Imran *et al.* (2023)^168^ developed an electrochemical sensor to accurately and quickly detect 5-hydroxymethylcytosine (5hmC) in genomic DNA. 5hmC is an epigenetic modification that plays a considerable role in gene regulation and has been implicated in various biological processes and diseases.

The researchers used a (G) transducer-based electrochemical sensor modified with carbon nitride to enhance the detection sensitivity and selectivity towards 5hmC. The carbon nitride modification on the (G) surface provided a stable and biocompatible platform for capturing and detecting 5hmC molecules. The article described the fabrication and characterization of the carbon nitride-modified (G) transducer and its application in detecting 5hmC. The electrochemical sensor demonstrated high selectivity towards 5hmC, distinguishing it from other cytosine derivatives and DNA bases. The sensor also exhibited excellent sensitivity, allowing for immediate detection of 5hmC with a low detection sensitivity.

**Fig. 12:**
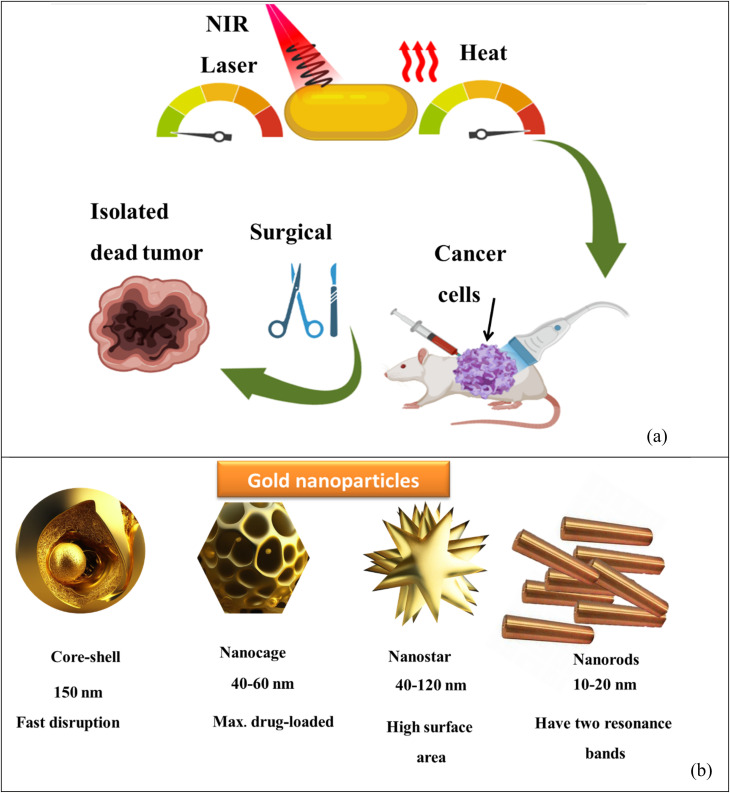
(a) In (PTT), nanoparticles are systemically delivered and accumulate within solid tumors through a phenomenon called the enhanced permeability and retention (EPR) impact. This impact exploits the leaky vasculature of tumors, allowing nanoparticles to accumulate in the tumor tissue selection. Once inside the tumor, (NIR) light is applied to the nanoparticles, causing them to generate heat through a process known as photothermal conversion. The generated heat is then used to select, ablate and destroy the tumor tissue while minimizing damage to the surrounding healthy cells. (b) Four of the most commonly used gold nanoparticles (AuNPs) for photothermal therapy.

The researchers further validated the performance of the electrochemical sensor by analyzing genomic DNA samples. They successfully detected and quantified 5hmC levels in genomic DNA, providing insights into its distribution and potential biological implications. Developing a highly selective and real-time electrochemical sensor for detecting 5hmC has noticeable implications in epigenetics research and biomedical uses. Accurate and sensitive detection of 5hmC can make an effort to improve comprehension of its role in gene regulation and various diseases, including cancer and neurological disorders. Fig. 12a illustrates Photothermal Therapy (PTT) principles, a promising approach in cancer treatment. In PTT, nanoparticles are systematically delivered and selectively accumulate within solid tumors, primarily exploiting a phenomenon known as the Enhanced Permeability and Retention (EPR) effect. This effect takes advantage of the leaky vasculature of tumor tissues, allowing nanoparticles to passively accumulate in the tumor.

Once these nanoparticles have accumulated within the tumor tissue, Near-Infrared (NIR) light is applied. When exposed to NIR light, these nanoparticles undergo photothermal conversion. During photothermal conversion, the nanoparticles absorb the NIR light energy and convert it into heat. This generated heat, localized within the tumor due to the accumulation of nanoparticles, is then harnessed as a therapeutic tool.

The heat generated in the process is precisely employed to selectively ablate and destroy the tumor tissue. Importantly, PTT aims to minimize damage to the surrounding healthy cells, making it a promising strategy for targeted cancer therapy. Fig. 12b provides a visual representation of four of the most commonly used gold nanoparticles (AuNPs) for photothermal therapy. These nanoparticles are pivotal in the PTT approach and have been extensively researched and applied in cancer treatment due to their exceptional photothermal properties. These illustrations and explanations in Fig. 12 provide a clear and concise overview of the fundamental principles and critical components of photothermal Therapy using gold nanoparticles, emphasizing the potential for selective and effective cancer treatment while sparing healthy tissues (Table 7).

**Table 7:** Compares four commonly used types of (GNPs) for photothermal therapy: nanocages, core–shell nanoparticles, nanorods, and nanostars

Aspect	Nanocages	Core–shell nanoparticles	Nanorods	Nanostar
Shape	Hollow, cage-like structures with porous walls	The core of one material (*e.g.*, Au) surrounded by a shell (*e.g.*, silica or polymer)	Elongated rod shape	Multiple branches or arms extending from a central core
Size range	Typically, larger sizes, ranging from 30–150 nm	Varied sizes depending on core and shell materials	Mixed sizes, but typically longer lengths (*e.g.*, 20–200 nm)	Diverse sizes, typically smaller than nanorods
Surface area	Relat larger surface area because of porous walls	Moderate surface area because of the core–shell structure	Moderate surface area	Somewhat larger surface area because of multiple branches
Plasmonic properties	Broad absorption spectrum across the visible and (NIR) region	Broad absorption spectrum across the visible and NIR region	Intense absorption in the NIR region	Strong absorption in the NIR region
Photothermal efficiency	High photothermal conversion efficiency	High photothermal conversion efficiency	High photothermal conversion efficiency	High photothermal conversion efficiency
Stability	Good stability in the solution	Good stability in the solution	Good stability in the solution	Good stability in the solution
Aspect ratio	Variable	Variable	1 : 3	1 : 1
Photothermal efficiency (%)	High (*e.g.*, 60–80%)	Moderate (*e.g.*, 30%)	High (*e.g.*, 70%)	Moderate (*e.g.*, 30%)

## 6 Respect future work

By addressing the following areas of future work, we can further expand our knowledge of (GNPs) optical features and their uses in diverse fields. The continued exploration of these fascinating nanomaterials contributes to advancements in nanotechnology, photonics, and materials science, ultimately leading to innovative technologies and solutions with noticeable societal impact.

(1) *Advanced characterization techniques*: advanced characterization techniques, like high-resolution electron microscopy, spectroscopy, and surface-enhanced Raman scattering (SERS), can provide more detailed insights into the structural and optical features of (GNPs). These techniques can help elucidate the underlying mechanisms governing nanoparticles' optical behavior and environmental interactions.

(2) *Size and shape control*: developing new methods for precise control over the shape and size of (GNPs) can open up opportunities for fine-tuning their optical properties. Exploring novel synthesis approaches, surface modification strategies, and template-assisted growth techniques may enable the production of nanoparticles with tailored optical responses for related uses.

(3) *Multifunctional nanoparticles*: integrating additional functional components, like plasmonic metals, semiconductors, or magnetic materials, into (GNPs) can lead to the development of multifunctional nanostructures with enhanced optical properties. Investigating the synergistic impact of combining various materials within a single nanoparticle could result in novel optical phenomena and enable the design of advanced devices for sensing, imaging, and energy-related uses.

(4) *Biomedical uses*: (GNPs) have shown great potential in various biomedical uses, including targeted drug delivery, photothermal therapy, and bioimaging. Further research is needed to optimize the design of (GNPs) for enhanced biocompatibility, stability, and specificity. Exploring their interactions with biological systems and understanding the underlying mechanisms pave the way for safer and more impactful uses in biomedicine.

(5) *Computational modeling and simulation*: the development of computational models and simulations can complement experimental studies and provide valuable predictive tools for understanding the optical behavior of (GNPs). By incorporating factors like particle size, shape, aspect ratio, and surrounding environment, these models can aid in designing and optimizing nanoparticles with desired optical properties.

(6) *Optimization of drug delivery systems*: although the reviewed studies demonstrated promising results, further optimization of drug delivery systems is warranted. This includes exploring novel materials, improving stability, enhancing drug loading capacity, and refining targeting strategies to increase specificity and reduce off-target impacts.

(7) *Combination therapies*: combining various therapeutic approaches has shown great potential in overcoming multidrug resistance and improving treatment outcomes. Future studies can focus on identifying optimal combination therapies, like chemo-photothermal therapy, to maximize synergistic impacts and minimize side impacts. Investigating various therapies' sequential administration and timing can further enhance their effect.

(8) *Mechanistic understanding*: gaining a deeper understanding of the underlying mechanisms involved in the therapeutic strategies is considerable for further development. Future research can explore the cellular and molecular mechanisms by which these therapies work, including the interactions among nanomaterials and cancer cells, drug release kinetics, and cellular uptake pathways. This knowledge can help refine the design and optimization of therapeutic approaches.

(9) *Clinical translation*: while the reviewed studies demonstrated efficacy in preclinical models, future work should focus on translating these findings into clinical uses. Conducting rigorous clinical trials to evaluate these therapies' safety, effect, and long-term outcomes in human patients is essential for their successful translation into clinical practice.

(10) *Personalized medicine*: the development of personalized treatment strategies based on individual patient characteristics, like tumor profiling and genetic markers, holds immense potential. Future research can explore integrating these personalized approaches with advanced therapeutic modalities to tailor treatment regimens to individual patients, maximizing therapeutic efficacy and minimizing adverse impacts.

**Fig. 13:**
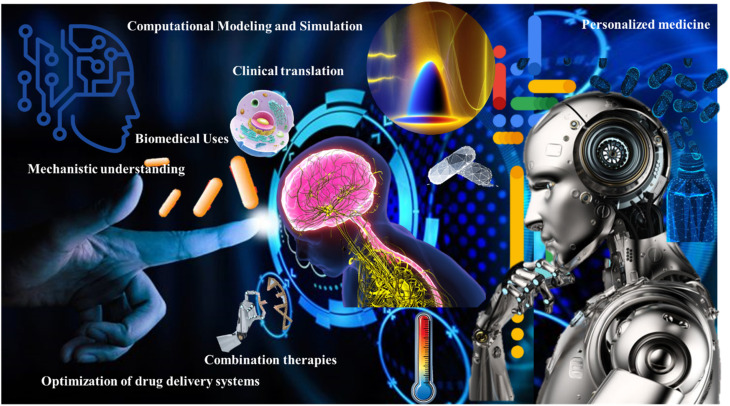
Emphasizes the dynamic and forward-looking nature of cancer research and therapy. It highlights the ongoing efforts to improve and refine treatments, ultimately striving for better outcomes and a brighter future in the fight against cancer.

Fig. 13 simulated future work to optimize drug delivery systems further, explore combination therapies, deepen the mechanistic understanding of therapeutic approaches, facilitate clinical translation, and advance personalized medicine approaches. These efforts contribute to developing more impact, targeted, and personalized cancer therapies with improved patient outcomes.

## 7 Conclusion

Gold nanoparticles' size, shape, and aspect ratio play considerable roles in determining their optical properties, specifically their surface plasmon resonance characteristics. By controlling these parameters, researchers can achieve precise optical tuning and tailor the nanoparticles' features for related uses. SPR bands change as gold nanoparticles transition from spherical to rod-like structures, aided by adding shape-modifying surfactants and reducing agents. The longitudinal SPR band exhibits a remarkable red-shift into the (NIR) region with increasing aspect ratios, while the transverse SPR band remains unchanged. Computational methods, like the discrete dipole approximation, provide valuable insights into gold nanoparticles' absorption, scattering, and total extinction features. Studies have shown that higher aspect ratios enhance the scattering efficiency and quantum yield, indicating increased light scattering capabilities. However, the absorption efficiency increases at higher aspect ratios, slightly decreasing the scattering quantum yield. These findings highlight the intricate interplay among size, shape, and aspect ratio in shaping the optical characteristics of gold nanoparticles. The ability to precisely tune these parameters opens up new possibilities for various uses, including biosensing, imaging, catalysis, and photothermal therapy. Continued research in this field further advances our understanding and utilization of gold nanoparticles for various optical and nanophotonic uses.

## List of abbreviation

AAO: Anodic aluminum oxide
ALG: Alginate
C16TAB: Surfactant
CRC: Colorectal cancer
CTAB: Surfactant
CUR: Curcumin
DCM: Dichloromethane
DDA: Discrete dipole approximation
DEPC: Diethyl pyrocarbonate
DMEM: Dulbecco's modified eagle medium
DOX: Doxorubicin
DPBS: Dulbecco's phosphate-buffered saline
DPL: Dip-pen lithography
DTX: Docetaxel
ELM: Extracellular matrix-like
EMT: Extended Mie theory
EPR: Enhanced permeability and retention
FDA: Food and drug administration
FEM: Finite element method
FIB: Focused ion beam
GNC: Gold nanoclusters
GND: Gold nanodiamond
GNP: Gold nanoparticles
GNR: Gold nanorods
GNS: Gold nanostar
IC_50_: Half maximal concentration
LIL: Laser interference lithography
LSPR: Localized surface plasmon resonance
MDR: Mediated multidrug resistance
MRI: Magnetic resonance imaging
MTT: (3-(4,5-Dimethylthiazol-2-Yl)-2,5-diphenyltetrazolium bromide)
NIR: Near-infrared-responsive
NLC: Nanostructured lipid carriers
OES: Optical emission spectrometry
PAH: Polyallylamine hydrochloride
PBS: Phosphate buffered saline
PDT: Photodynamic
PEG: Polyethylene glycol
PEI: Polyethylene imine
PHIS: Ph-responsive poly-histidine
PLA: Poly lactic acid
PPT: Photothermal therapy
PPTT: Plasmonic photothermal therapy
PSS: Polystyrene sulfonate
PTX: Paclitaxel
ROS: Reactive oxygen species
SDT: Sonodynamic therapy
SPION: Superparamagnetic iron oxide nanoparticles
SPR: Surface plasmons resonance
TPL: Two-photon excited luminescence

## Conflicts of interest

There are no conflicts to declare.

## Supplementary Material
